# Photoinduced Transport and Activation of Polymer-Embedded Silver on Rice Husk Silica Nanoparticles for a Reusable Antimicrobial Surface

**DOI:** 10.3390/nano15161224

**Published:** 2025-08-11

**Authors:** Carly J. Frank, Vivian He, Juan C. Scaiano, M. Jazmin Silvero C.

**Affiliations:** Department of Chemistry and Biomolecular Sciences, University of Ottawa, Ottawa, ON K1N 6N5, Canada; cfran081@uottawa.ca (C.J.F.); vhe012@uottawa.ca (V.H.)

**Keywords:** antibacterial, silver nanostructures, rice husk, photoactivation

## Abstract

Antimicrobial materials are gaining significant interest as awareness of pathogens spread through contact becomes increasingly prevalent. While various compounds with antibacterial properties have been explored as active ingredients in such materials, many are prone to leaching, leading to undesirable risks to the environment and to human health. Herein, we develop and test a multilayered plastic film filled with silver nanoparticles, long known to be potent antibacterial agents, supported in a silica matrix. Cross-linked methacrylate layers on both sides of these nanostructures prevent leaching even after several uses, making the material essentially benign. Furthermore, we derive silica from rice husk, an abundant and affordable agricultural waste product. Our findings demonstrate that initial irradiation of the material with UVA light facilitates the photothermal migration of nanoparticles towards the material’s surface, thereby significantly enhancing its antimicrobial properties. Remarkably, after just 5 min of visible light irradiation, the material exhibits over 99.999% inhibition of bacterial growth. This environmentally friendly plastic composite harnesses visible light to actively combat bacteria, providing an exciting proof-of-concept for future applications in antimicrobial coatings.

## 1. Introduction

The constant presence of fomites in our daily lives poses a significant challenge to public health. As we interact with common surfaces like doorknobs and communal tables, the potential for widespread pathogen transfer is staggering and constant. Children aged 0–2 years are particularly vulnerable to this risk. This developmental stage involves extensive exploration of their environment through touch and frequent mouthing of objects and surfaces in shared spaces like childcare facilities. This inherent hand-to-mouth behavior significantly amplifies their susceptibility to fomite-mediated infections. Children not only touch contaminated objects but also repeatedly put them into their mouths, directly introducing pathogens into their systems [1].

Frequent interactions transform everyday objects into silent vectors, contributing significantly to the global burden of infectious diseases. These vectors facilitate the invisible spread of pathogens from hand to surface and back again, posing a major concern [2]. The risk of transmission is further amplified by the high frequency of contact with common fomites, prompting numerous efforts to develop cleaning and disinfection agents. However, most commercial disinfectants rely on harsh chemical compounds, such as quaternary ammonium compounds and chlorine-based agents, which pose recognized risks to both human health and the environment [3]. These compounds can lead to respiratory issue and skin irritation, and even have potential links to asthma and reproductive problems [4]. They also form toxic byproducts and disrupt aquatic ecosystems [5]. For instance, n-nitrosamines, chloroform, bromodichloromethane, dibromochloromethane, and bromoform are known to be possible human carcinogens. Once these compounds are leached into the water, further treatment is usually needed to generate subsequent byproducts that are less toxic. In response to these concerns, antimicrobial plastics have emerged as an intriguing option [6,7]. However, they often contain additives like triclosan or small spherical silver nanoparticles (AgNP) that are not bound to the polymer matrix and can leach into the environment. Triclosan has been associated with endocrine disruption, developmental toxicity, and the promotion of antimicrobial resistance. Leached silver nanoparticles, on the other hand, can be highly toxic to aquatic organisms, disrupting microbial communities and potentially accumulating in the food chain [8].

In this work, we aimed to create an intrinsically photo-active antibacterial polymer that could be used as a coating or rigid material in self-cleaning products. Unlike conventional disinfectants that rely on chemicals or hazardous agents, this material would inhibit microbial growth when exposed simply to visible light, eliminating the need for further intervention. Our system comprises three key components: a rigid cross-linked polymer matrix, photo-active silver nanoplates, and naturally derived nano silica as support. The silver–silica nanoparticle layer is expected to act as the active species. While rice husk-derived silica (RHsil) has been studied previously and found to be an effective component for similar goals including antimicrobials and degrading organic pollutants in water [9,10,11], such studies tend to focus on the material in its powder form. To encourage the shift from the lab scale to the application of this material, we focus herein on encapsulating the nanoparticle mixture in a rigid polymer while maintaining its anticipated antimicrobial capabilities.

An important discovery made during this research is that the antibacterial properties of these polymer-embedded silver on rice husk silica nanoparticles (AgNP@RHsil) can be significantly enhanced by exposure to UVA light. This enhancement is unidirectional, meaning it only occurs on the surface directly exposed to the light source. We propose that a photothermal mechanism is behind this enhancement, causing the polymer to soften and facilitate material transport towards the light source. This effect is reminiscent of phototropism, which causes plant growth towards a light source [12].

## 2. Results

### 2.1. AgNP@RHsil Nanoparticles Synthesis and Inclusion in Polymer Matrix

**Figure 1:**
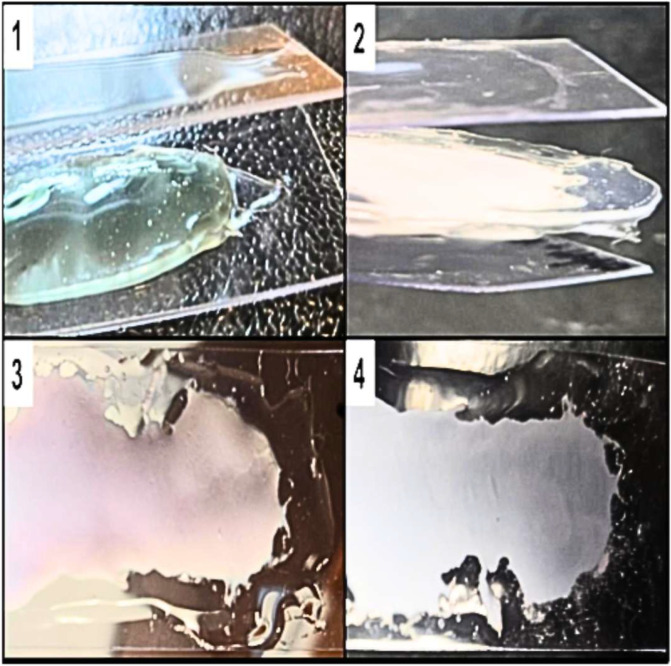
(**1**) Side view of sandwich preparation showing the bottom solidified gel layer with the light blue slurry of nanoparticles and the second microscope slide with gel to be cross-linked on top; (**2**) side view while peeling off the microscope slides to obtain the multilayered material after UVA exposure; (**3**) top view of the material (light purple) and (**4**) bottom view (white).

The procedure to obtain SiO_2_ nanoparticles from rice husk (RH) produces a fine white powder that turns blue after mixing with AgNP. Interestingly, the powder regains its white color when air-dried. To create a protective multilayered structure—similar to a sandwich—a polymer film was applied to a plastic microscope slide, irradiated under UVA to encourage cross-linking, and then a slurry of nanoparticles was added on top. Another layer of polymer was applied to a second microscope slide, and the sandwich-like arrangement was assembled by placing the top layer on the bottom layer, with the nanoparticles serving as the filling. UVA irradiation of the upper layer was necessary to cross-link and solidify the polymer. After UVA irradiation of the upper polymer layer for cross-linking, the color of the top of the sandwich was observed to be light purple, while the bottom remained white (Figure 1). This phenomenon was further investigated using TEM/SEM imaging. When the UVA source lamp was used for 10 min on the surface of the microscope slide containing the AgNP@RHsil sandwich, the temperature reached 55 °C, while it remained at 26 °C for the control samples without the nanoparticles. While 55 °C was the exterior temperature of this plastic composite, it is likely that the temperature in the immediate vicinity of the AgNP was significantly higher, thus achieving a significant softening of the polymer matrix.

### 2.2. AgNP@RHsil Nanoparticles Characterization Without and Within the Polymer

**Figure 2:**
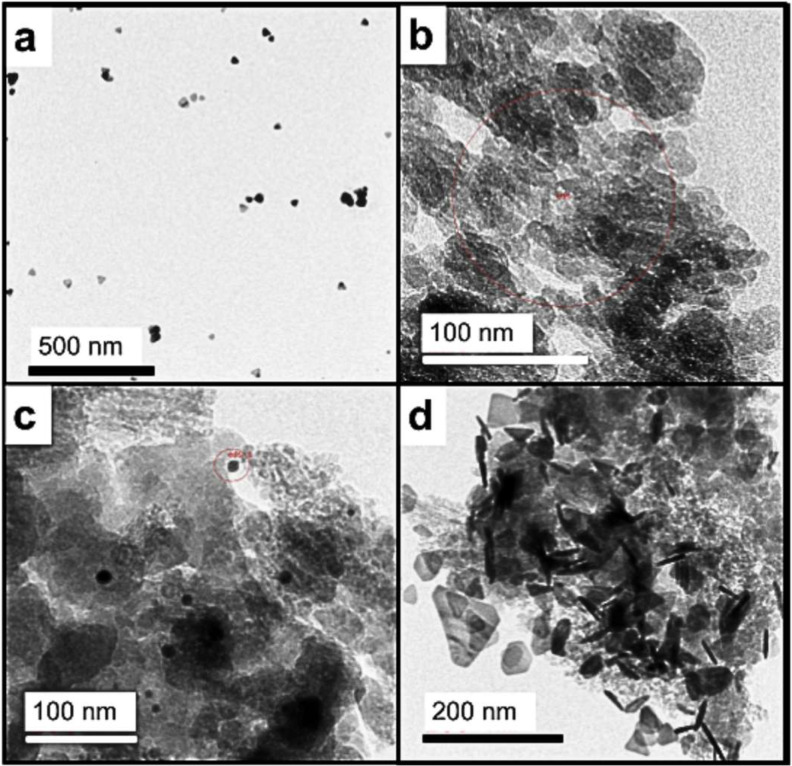
Selected TEM images (cropped) of: (**a**) tAgNP (triangles synthesized in flow and washed); (**b**) RHsil (silica extracted from rice husk); (**c**) AgNP@RHsil nanoparticles after mixing 1 + 2 and washing; (**d**) AgNP@RHsil nanoparticles after UVA exposure (just the powder, no polymer).

AgNP synthesized in flow [13] were deposited onto a copper grid and imaged by TEM (Figure 2a). Some intermediate morphologies were visible; however, the majority of the species resembled triangular plates as expected.

RHsil nanoparticles were typically small and round, and some striation-like ordering could be seen in some TEM images (Figure 2b). It has been noted before that, like cellulose, bio-derived species retain some “memory” [14,15] of the well-ordered plant structure. We believe that this is what is observed here. AgNP@RHsil were imaged before and after UVA exposure, and changes in the surface particles were clearly visible. Prior to exposure, the AgNP visible to the TEM were dispersed across the RHsil matrix (Figure 2c). Following UVA exposure, larger quantities of AgNP triangles appeared (Figure 2d). Full EDS report examples (EDS areas marked by red circles in selected SEM images) can be found in the Supplementary Material, Figures S6 and S7.

**Figure 3:**
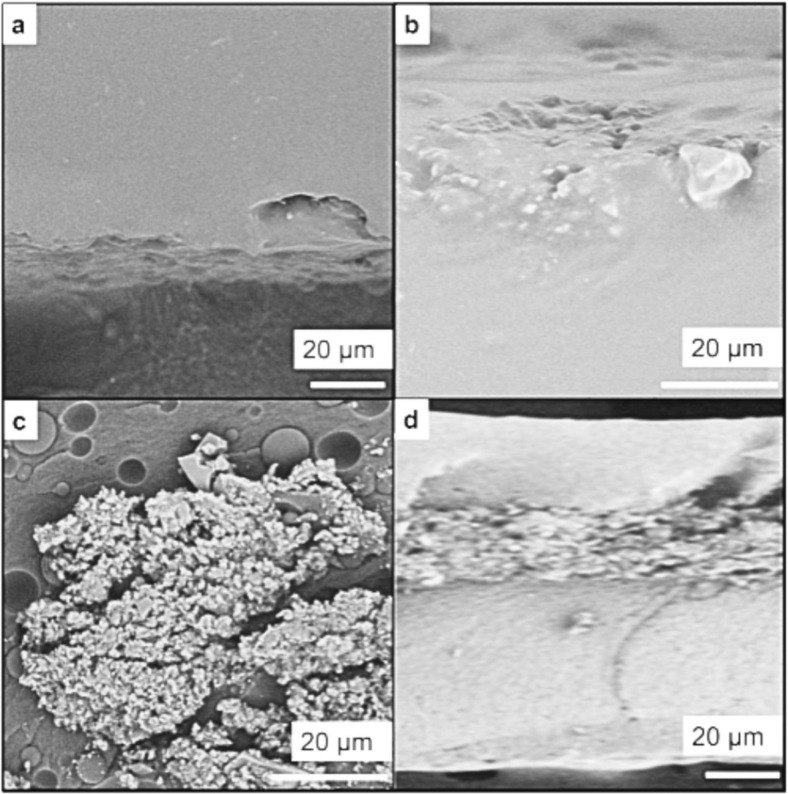
Selected SEM images of cross-sections of the material (cropped): (**a**) bottom layer (polymer alone); (**b**) top layer (polymer with nanoparticles close to the surface); (**c**) middle layer (filling of AgNP@RHsil); (**d**) side view of the three layers. Uncropped images can be found in Supplemental Information (Figures S11–S13).

In contrast, SEM analysis of cross-section slides of the multilayered material revealed the migration of nanoparticles after the photoinduced cross-linking of the second polymer layer. The AgNP@RHsil filler particles were found closer to the upper surface. The top layer was thinner than before irradiation and appeared partially merged with the middle layer. The bottom layer (that had been cross-linked initially) remained smooth and nanoparticle-free (Figure 3).

**Figure 4:**
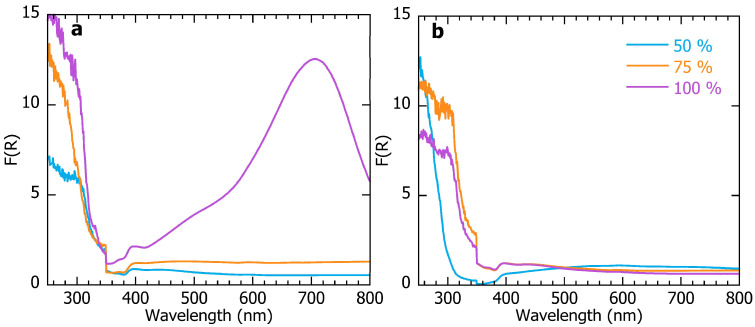
Diffuse reflectance spectra of (**a**) the top and (**b**) the bottom of multilayered material samples cross-linked by the Kessil lamp at 50% (blue), 75% (orange), and 100% (purple) of its maximum power.

To confirm the observations above, DRS measurements were also performed on samples of the material which had been cross-linked at different power settings under the Kessil lamp. Both the top layer of the material (the side exposed to the lamp in the final crosslinking step, after the nanoparticle slurry had been applied) and the bottom were measured. These spectra, shown below, demonstrate that nanoparticles were more easily detected at the top layers with increasing irradiation (Figure 4).

### 2.3. Antimicrobial Capacity of AgNP@RHsil Without and Within the Polymer

**Figure 5:**
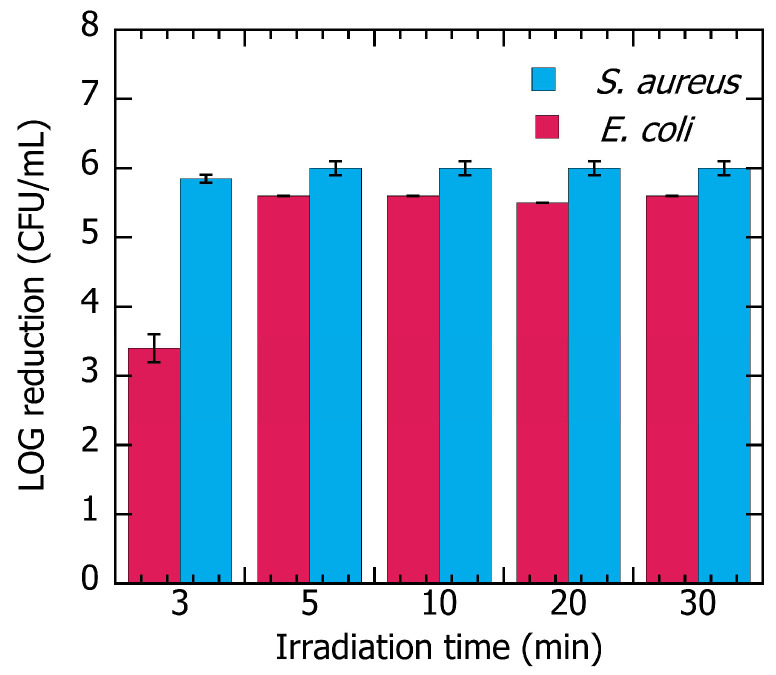
Log reduction in *E. coli* (pink) and *S. aureus* (blue) on the surface of the multilayer material at different irradiation times under visible light. Duplicates kept in darkness and the controls over polymer alone, and sandwiches filled with non-migrated AgNP@RHsil, white rice husk ashes, black rice husk ashes, and silver triangles nanoplates alone did not show significant modification in bacterial growth at any time (LOG reduction < 0.1) and were not included in this graphic. Results for the eight independent experiments using *E. coli* are available in the Supplementary Material, Table S1.

Nanoparticles suspended in solution completely inhibit the growth of *Escherichia coli* after irradiation for 60 min or in darkness for 180 min. This is likely due to the inherent antibacterial properties of both Ag and SiO_2_ nanoparticles. The multilayer polymer including AgNP@RHsil showed much more promising results, reducing the *E. coli* growth by 5 Log units (99.999% inhibition) after being irradiated with white LED panels for 5 min, and by 3 Log units (99.9%) after just 3 min of white LED irradiation (Figure 5). A Gram-positive strain, *Staphylococcus aureus*, was also tested in the same manner to assess if the nanoparticles still preserved their broad spectra of action. These results (Figure 5) were very positive, with a 5 Log unit inhibition of *S. aureus* growth after 3 min, and a 6 Log reduction (99.9999%) after 5 min of white LED irradiation.

The enhanced antimicrobial performance and significantly reduced inhibition times may stem from the strategic positioning of the nanoparticles within the multilayered architecture of the material (Supplementary Material, Figure S17). This configuration maximizes their exposure to visible light and facilitates closer contact between the nanoparticles and microorganisms, aided by the photoinduced transport mechanism described earlier. Notably, this surface did not show any inhibition in the dark (Supplementary Material, Figure S16), indicating that the nanoparticles were not directly exposed or released to the bacterial inoculum. This antibacterial efficacy was maintained for at least eight consecutive uses when tested against *E. coli* (Table S2).

**Figure 6:**
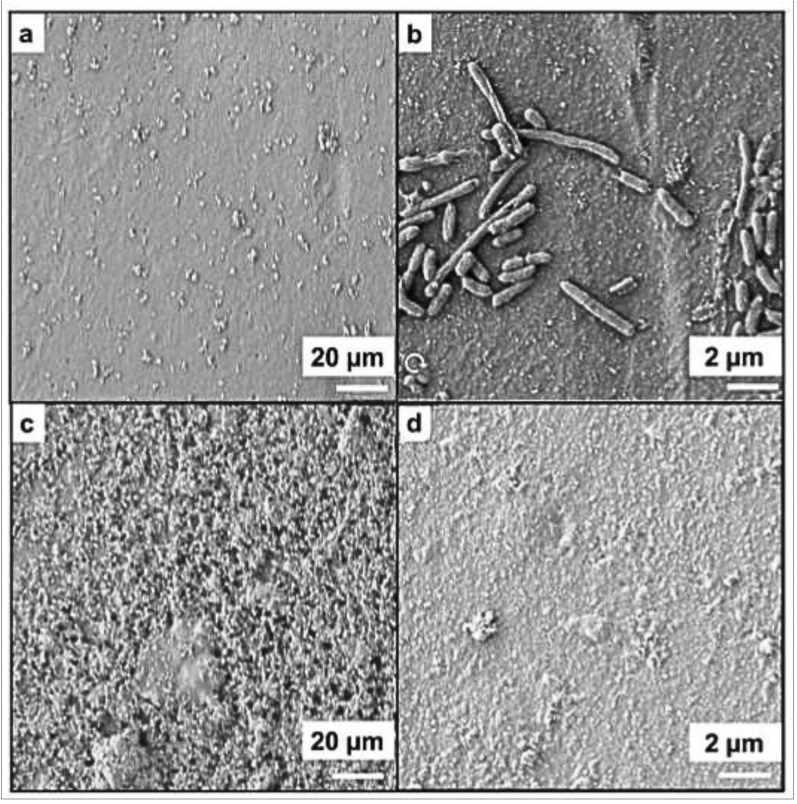
Selected SEM images (top view) of multilayered material containing AgNP@RHsil (cropped): (**a**) before UVA cross-link irradiation; (**b**) exposed to bacteria inoculum of *E. coli* and kept in darkness for 10 min showing bacterial growth; (**c**) after cross-linked irradiation showing the migration of nanoparticles to the surface; (**d**) exposed to bacteria inoculum of *E. coli* and irradiated with white LEDs for 10 min showing the absence of bacteria.

SEM images of the surfaces of the layered structure (under irradiation and darkness) and of control samples with only the polymer layer are consistent with the CFU counting results, confirming that no intact bacteria could be found after 10 min of irradiation (Figure 6). Only a few fragments of bacteria membranes were found after exposing entire pieces (duplicates) to light, while groups of bacilli could be easily observed in the controls. This bacterial debris represents under 2% of the total surface area in one of the analyzed pieces (1 cm × 1 cm); the other sample had no visible debris. Between 40 and 50 SEM captures of each sample were analyzed with Fiji software (version 2.9.0) [16] to determine this value accurately. Controls of bacteria on top of the polymer alone were fixed and treated in the same way to ensure that the microorganism was not detaching from the surface of interest.

The control test involving bacteria inoculum on the polymer alone as mentioned above also represents the control experiment for temperature to confirm that the system’s bacterial growth inhibition is due to a photothermal mechanism, for which the nanoparticle layer is a key component. Indeed, there was no discernable damage to the microorganism’s integrity in this sample (Supplementary Material, Figure S18). This suggests that both the proximity of nanoparticles to the surface and light irradiation are essential for achieving the antimicrobial effect.

### 2.4. Leaching Tests

ICP measurements showed there was no significant presence of Ag or Si in the collected water samples run through the freshly made polymer surface (0.08 ppm) when used one time (0.08 ppm) and used eight times (0.07 ppm). The calibration curve and control with nanoparticles detected Ag and Si in as low as 0.01 ppm and up to 50 ppm.

## 3. Discussion

In this work, we investigated the potential of tAgNP (triangle silver nanoplates), which have recently shown remarkable photo-antimicrobial properties [17], to develop a polymer suitable for use on fomite surfaces. Silver has been widely used as an antimicrobial agent for a long time, finding applications in various fields and industries including medicine, food packaging, water treatment, and cosmetics [18,19,20]. Recent research has revealed that the morphology and dimensions of AgNP are highly tunable, and that the wide range of morphologies enables modification of their optical and electronic properties [21]. Our research group has found that photochemically generated platelike silver triangles outperform both spherical “seeds” and decahedral AgNP in antibacterial applications [17]. Additionally, the color-controlled synthesis of AgNP serves as a powerful tool to scale up the production of custom silver morphologies [13,17]. To transition this study toward a solid-phase material, tAgNP should be incorporated onto a suitable support that prevents oxidation and unwanted loss, while maintaining sufficient surface proximity to preserve their antimicrobial efficacy [22]. For this purpose, we explored the use of rice husk-derived silica as a support matrix and methacrylate cross-linked polymer as a material to contain them.

As the concept of green chemistry gains momentum, more effort is being placed on finding bio-inspired or naturally derived materials. Many of these materials possess novel characteristics not found in typical support materials, giving rise to several decontamination applications across inorganic, organic, and bacterial species [9,23,24]. Rice husk (RH), a byproduct of rice production and processing, is often considered agricultural waste [25]. However, recent reports have shown its potential as a support material for heterogeneous catalysis due to its high silica content [26]. This, coupled with its abundance and affordability, makes it an attractive option. Further, research has shown that RH silica (RHsil) exhibits a large surface area exceeding 300 m^2^/g and pore sizes around 5 nm, while retaining some of the macroscopic features of the original husk [27].

RHsil was easily combined with AgNP using APTES as a binding molecule [28]. Once the AgNP are embedded in the SiO_2_ matrix, they tend to remain isolated within the pores, more so than they would in a liquid suspension. This isolation could explain the apparent loss of blue color. Additionally, silica helps prevent aggregation and enhances the long-term stability of the AgNP (Figure 2).

Methacrylate polymers, commonly found in nail gels, are well-suited for photo crosslinking with UVA light due to their chemical structure and the presence of specific photo initiators, such as benzophenone [29]. The free radicals generated by the photoinitiated attack the carbon–carbon double bonds of the methacrylate monomers and oligomers, initiating a chain reaction. The polymerization process terminates when two growing radical chains combine or when a radical reacts with an impurity or oxygen. This process rapidly increases the molecular weight and leads to the formation of a highly cross-linked, durable, and generally inert polymer network [30]. This makes them highly interesting for incorporating active antimicrobial agents like nanoparticles [12,31]. However, sometimes these additives can interfere with the optimal photo-polymerization. Indeed, the first attempt to include the AgNP@RHsil directly into the polymer gel in different ratios did not work well. The material remained sticky even after long periods of UVA irradiation, indicating that the AgNP@RHsil inclusions were preventing efficient cross-linking of the polymers.

In our second approach, we developed a sandwich-like multi-layered material, consisting of two layers of polymer filled with nanoparticles. With this method, methacrylate chemistry is not hindered by the nanoparticles during the cross-linking process. At the same time, photothermal effects due to UVA absorption by the nanoparticles from the filling promotes their migration to the surface, as indicated by SEM imaging of a cross-section of the material (Figure 3) and by DRS data, wherein the characteristic spectrum of tAgNP in the visible region became clearer with increasing light intensity on the top, and conversely decreased in the region on the bottom of the same sample. The temperature of the material reached 55 °C during this process as measured using an IR thermometer (Etekcity Lasergrip 800, Anaheim, CA, USA). The resulting cured gel of the upper layer of this smart-designed material looked like the melted cheese over the toppings in a pizza. We playfully refer to this observation as “the sandwich that turned into a pizza”.

Evidence supports that the nanoparticles also underwent some morphological and location changes after UVA irradiation of the upper polymer layer to cross-link the methacrylate. In fact, this agrees with previous findings about the continuous increase in the Ag nanoparticles (in number and size) with the irradiation time [32]. Measurements of the irradiance of the Kessil lamp through the plastic microscope slides used as support in this synthesis indicate that both a small amount of UVA, as well as a visible light emission from the slide itself, reach the material (Figure S18). We propose that this combination simultaneously enables the cross-linking of the polymer as well as the photo-activation of RHsil and AgNP, which have strong absorptions in the UVA and in the visible region, respectively. This irradiance can also impact the morphological characteristics of the AgNP, changing their shape and optical properties. Specifically, the sharp corners of the triangles are unstable, and upon UVA and short-wave visible light irradiation, atoms migrate to form more rounded, stable shapes [33]. This process, sometimes called “backward tuning,” allows for the creation of new shapes. Atoms from these corners migrate to the flat surfaces of the triangle, causing the sharp edges to round off and the overall shape to become more circular. As the shape of nanoparticles change, so do their optical properties. This includes a shift in their plasmon resonance. While the exact mechanism is still being researched, it is believed that the migration is driven by the higher energy and instability of the corner atoms compared to those on the flat surfaces [34,35]. Given the characteristic blue color of tAgNP aqueous solution, this photoinduced transportation and new distribution could explain the color change from white to light purple. TEM images taken after this step showed that the silver tAgNP were now more exposed to the surface of the matrix and some other morphologies were found.

Consistent with these observations, SEM images of cross-sections of the resulting layered material showed that the nanoparticles were not retained in the middle of the two polymer layers. Rather, they migrated to the upper layer and were now just sufficiently close to the surface to perform their antimicrobial photo-activated effect, but not enough to leach out of the material thanks to the upper cross-linked polymer. We attribute the migration to the known behavior of silica within polymers when exposed to UV [36]. In fact, it was reported that UV can bring silica to the surfaces of epoxy materials where they were contained, and an excess can lead to their release. The dose of UVA light used in this case is just enough to bring them close to the surface (Figure 6c).

We tested the material antimicrobial capacity starting with *E. coli* because it is among the most spread microbes and it is the second most common cause of death linked to bacterial infections globally. Different strains of this pathogen are the leading cause of community-onset sepsis and bacteremia, affecting millions of patients worldwide each year [37,38]. Inhibition of growth seems to be achieved only when the multilayered material is irradiated with visible light, leading to the conclusion that the most plausible mechanism of inactivation is photothermal [39,40,41] (Figure 5). This mechanism is also supported by the ICP-MS measurements of water samples that suggest no leaching of either silica or silver at all (Supplementary Material, Table S2). Duplicates kept in darkness and the controls over polymer alone, as well as the sandwiches filled with non-migrated AgNP@RHsil, white rice husk ashes, black rice husk ashes, and silver triangles nanoplates alone did not show significant modification in bacterial growth at any time, even under light (LOG reduction < 0.1). This finding indicates that clearly, there is not an effect of direct heating from LED panels. On the other hand, the fact that this material is inhibiting the growth of both *E. coli* and *S. aureus* through a photothermal mechanism (triggered by the AgNP@RHsil close to the surface) is a strong indication that this antimicrobial effect is not microbe-specific. We demonstrated previously that this increase in the local temperature on the surface of the metal nanoparticle can lead to irreversible damage on the bacteria membrane independently of their antibiotic resistance mechanisms [42]. The results in both Gram-negative and Gram-positive bacteria suggest that the multilayer material is a potential broad-spectrum antimicrobial agent. Further studies on different bacterial strains and even temperature resistant spores are planned to expand the application of this material in the prevention of fomite-mediated infections [43,44].

This novel material now has several advantages due to the high cross-link density of the polymer and the optimized location of the antimicrobial nanoparticles. First, the interconnected polymer chains form a rigid structure resistant to deformation and chipping. Furthermore, the material also has good chemical resistance, as the tightly bound network would be minimally susceptible to dissolution in, or uptake of common solvents. Repeated tests on the same samples also indicate a high degree of durability and reusability, with antimicrobial activity being retained throughout several uses. In addition, very low, if any, leaching was detected due to the high degree of polymer cross-linking to protect and encapsulate the nanoparticles. This enclosure of nanoparticles gives rise to photothermal inhibition of bacteria growth, making their antibacterial effect broad and capable of discouraging the growth of any temperature-susceptible microbe in contact with the surface, regardless of the microbe’s membrane composition.

## 4. Methods and Materials

### 4.1. Materials and Equipment

Silver nitrate was obtained from Alfa Aesar (Alfa Aesar Ward Hill, MA, USA),while trisodium citrate dihydrate was purchased from Fisher Scientific (Fisher Scientific, Hampton, NH, USA). Irgacure-2959 was provided by BASF chemicals. Rice husks were purchased from Toronto Brewing (Toronto Brewing, Toronto, ON, Canada). Makart nail extension gel, a mixture of acrylates copolymer, polyurethane, ethyl(2,4,6-trimethylbenzoyl)-phenylphosphinate, and silica dimethyl silylate, was purchased for use as the acrylic polymer. Hydrochloric acid was obtained from Fisher Scientific (Fisher Scientific, Hampton, NH, USA), and Luria–Bertani (LB) broth and Mueller Hinton (MH) agar were purchased from Sigma Aldrich (Sigma Aldrich, St. Louis, MO, USA) and BD Life Sciences(BD Life Sciences, Franklin Lakes, NJ, USA), respectively. All water used was ultrapure, obtained through the purification of deionized water using a Thermo Scientific Barnstead GenPure water purification system (Madison, WI, USA), achieving a conductivity of 18 MΩ cm^−1^. Plastic microscope slides (Rinzl brand, Burgaw NC, USA) of 3” × 1” were used in the material preparation as a base and then peeled off. *Escherichia coli* 25922 and *Staphylococcus aureus* 25923 inocula were grown periodically from frozen stocks.

The light sources and expo panels for silver nanoparticle synthesis were obtained from Luzchem Research (Luzchem Research, Ottawa, ON, Canada). Teflon tubing for wrapping the lamps was purchased from Component Supply, and a Gilson MiniPuls 3 (Gilson, Madison, WI, USA) provided a suitable range of flow rates for both fast-pumped seed synthesis and slow-pumped AgNP conversion.

Antimicrobial experiments were conducted on the finished layered materials using two SOLLA 150 W white LED flood lights (400–700 nm, made in China), which produced a maximum light irradiance of 284 W/m2.

Electron microscopes used were from Carleton Nano Imaging Facility: Tescan VegaII XMU SEM (Tescan Group, Khoutovice, Czech Republic) and FEI Tecnai G2 TEM (Thermo Fisher, Waltham, MA, USA)done our best, each with EDX capability. Some SEM pictures were acquired in uOttawa’s Materials Characterization Facility (MatChar, Ottawa, ON, Canada). UV-visible and Diffuse Reflectance spectra were taken in an Agilent Cary 7000 (Agilent Technologies, Santa Clara, CA, USA)

### 4.2. AgNP@RHsil Nanoparticles Synthesis and Inclusion in the Polymer

#### 4.2.1. AgNP Synthesis in Flow

AgNP seeds were synthesized using a flow system as previously described by our group [13,45]. Briefly, an aqueous solution containing 0.2 mM silver nitrate, 1 mM trisodium citrate, and 0.2 mM I-2959 was flowed through Teflon tubing (ID = 1.5 mm) doubly wrapped around a UVB expo panel lamp. This process resulted in the formation of a yellow solution with a characteristic absorbance at 400 nm, typical of AgNP seeds. The residence time for this step was approximately 4 min. Gentle cooling was applied to the unit exterior using a fan during this transformation.

To transform AgNP seeds into tAgNP, a series of warm white, amber, and red LED expo panel lamps were connected [13]. Silver nanoparticle seeds were gradually pumped through Teflon tubing, wrapping the lamps to produce a blue solution with a maximum absorbance around 700 nm. A sheet of aluminum foil was used to cover the flow lamps for both seed and tAgNP synthesis, reflecting light back into the system. Prepared nanoparticle solutions were stored in glass vials in the dark and were used as prepared.

#### 4.2.2. RH Preparation and Purification

The procedure was optimized from the literature [33]. First, 30 g of RH were ground into fine powder using a coffee grinder. The powdered RH was then subjected to multiple washing cycles to remove impurities. Each cycle involved adding 25 milliliters of distilled water, sonicating for 10 s, and centrifuging at 1000 rpm for 2 min until the supernatant became clear. The washed RH powder was then dried at 100 °C.

Next, 20 g of dried RH powder underwent acid treatment to remove organic components. This was achieved by stirring the powder in 40 milliliters of 5 M HCl at room temperature overnight. The resulting dark brown mixture was then vacuum-filtered, and the solid residue was thoroughly washed with distilled water.

#### 4.2.3. Silica Extraction and Functionalization

The acid-treated RH powder (14.5 g) was then calcined at 600 °C for 2 h in a muffle furnace. This step transformed the brownish powder into a white material, indicating a high silica content, and yielded 13.7 g of RHsil. (Supplementary Material, Figure S1).

The RHsil was then functionalized to enable attachment of silver nanoparticles. Silica (13.7 g) was dispersed in 80 mL of methanol, and 6 mL of (3-Aminopropyl)triethoxysilane (APTES) was added. This mixture was stirred overnight at room temperature and then stirred for 1 h at 70 °C. The resulting functionalized RHsil was then washed once with methanol and twice with distilled water. Finally, it was spread on a tray and air-dried at room temperature, yielding 13 g of functionalized silica particles.

#### 4.2.4. Preparation of AgNP Embedded on RHsil (AgNP@RHsil)

To create the AgNP@RHsil nanoparticles, 13 g of functionalized RHsil were combined with 130 mL of the tAgNP suspension described earlier. This mixture was stirred for 10 min. Subsequently, the desired AgNP@RHsil nanoparticles were isolated by centrifugation at 2000 rpm for 5 min, followed by two washes with ultrapure water to remove any unreacted materials (Supplementary Material, Figure S2).

#### 4.2.5. AgNP@RHsil Inclusion into a Polymer Film

Nanoparticles were incorporated into methacrylate-based gel through two distinct methods. The initial method involved directly adding the nanoparticles to the gel and then exposing it to UVA irradiation to cross-link the polymer chains and solidify the mixture (Supplementary Material, Figure S3). In contrast, the second method, which was eventually preferred, involved preparing three layers in the sandwich-like manner as described earlier, with layers of gel being cross-linked separately (Supplementary Material, Figure S4). For cross-linking, irradiation was always performed using a Kessil lamp (Kessil, Richmond, CA, USA) at 100% power for 10 min placed at a distance of 2 cm (Supplementary Material, Figure S5).

### 4.3. Characterization of AgNP@RHsil Without and Within the Polymer

The nanoparticle-containing layered materials and their individual components were characterized using Diffuse Reflectance (DR) and transmission electron microscopy (TEM) coupled with energy dispersive spectroscopy (EDS). The sandwich-like material was imaged using scanning electron microscopy (SEM) with EDS (Supplementary Material, Figures S6–S15). The samples for TEM were prepared by suspending the AgNP@RHsil powder in ultrapure water and dropping 10 μL onto a copper grid. The samples for SEM were obtained by slicing the sandwich-like materials to obtain fine cross-sections and then coating them with 10 nm of gold.

### 4.4. Antimicrobial Capacity of AgNP@RHsil Without and Within the Polymer

Bacterial cultures for antimicrobial susceptibility testing were prepared according to the Clinical and Laboratory Standards Institute (CLSI M100) guidelines [46]. Briefly, defrosted *E. coli* suspension was subcultured onto Mueller Hinton agar media and incubated at 37 °C for 18–24 h to obtain isolated colonies. A bacterial suspension of 108 CFU/mL was then prepared in sterile PBS, adjusted to a 0.5 McFarland standard. Serial dilutions were made to obtain a 104 CFU/mL final suspension that emulates the most frequent maximum surface contamination load and was used within 15 min.

The mixed polymer with AgNP@RHsil was tested by placing equal amounts (2 mg) at the bottom of the wells, followed by cross-linking with UVA. Then, 100 μL of the bacteria inoculum was added. Controls using RHsil, AgNP, polymer, and PBS alone were included. Two identical well plates were prepared, one kept in darkness and the other irradiated under the white LED panels described. Aliquots (10 μL) were taken from each well at t = 0, 60, 120, and 180 min and diluted properly to seed them in agar plates for CFU counting after overnight incubation at 37 °C. Triplicates were run, and results were expressed as the average.

The sandwich-like material underwent testing following the ISO 21296 procedure recommended for solid potential antimicrobial surfaces [47], with minor modifications to better replicate real-life conditions. Briefly, 1 cm × 1 cm pieces were cut in sterile conditions and placed inside empty Petri dishes. A drop (200 μL) of bacterial inoculum was added on top of each piece, and the Petri dishes were carefully capped and placed under the same visible LED panels for irradiation (Supplementary Material, Figure S15). Aliquots were taken after 3, 5, 10, 20, and 30 min, placed next to a flame, and seeded in agar plates for further incubation and counting of CFUs. The control set was kept in darkness. Duplicates were run for all conditions, and results were expressed as average. The experiment was conducted eight times to assess the material’s re-usability. Throughout the experiment, the temperature was monitored using an infrared thermometer in the vicinity of the Petri dishes and on the surface of the pieces at the end of the experiment.

### 4.5. Leaching Test

The release of nanoparticles was evaluated according to a technical report suggested by the International Organization of Standardization [48]. Briefly, ultrapure water flashed through the surfaces of the samples at room temperature (100 mL at the time, 20 times each). Then, collected samples were left to dry inside an incubator at 37 °C. Prior to ICP, all samples were digested for 1 h with 0.4 mL of concentrated HNO_3_ and 1.2 mL of concentrated HCl. Then, the suspension was heated overnight at 75 °C until the solid was completely dissolved, and the total volume of the sample decreased to about 0.5 mL. Next, 8 mL of ultrapure water was added, and the solution was transferred to a 15 mL Falcon tube to be centrifuged (10,000 rpm, 10 min). Finally, supernatant was collected in another Falcon tube and ultrapure water was added quantitatively to complete 10 mL. Triplicates were run and control was made with 10 mg of AgNP@RHsil.

## 5. Conclusions

We designed and tested a material incorporating AgNP@RHsil which demonstrated durable, reusable antimicrobial activity driven by the photo-activated properties of SiO_2_ and AgNP. This proof-of-concept offers an exciting range of possible applications in manufacturing and surface coating, particularly for products that are prone to contamination and challenging to clean due to frequent use. Potential examples include public doorknobs and handles, diaper changing stations, children’s toys, and playground equipment, all of which are regularly exposed to white light. The precise cross-linking polymer network yields a rigid, robust structure which does not leach, protecting the environment from undesirable chemicals, and which can be reused several times without losing antibacterial activity. The synthesis process is also reasonably green, working in aqueous conditions and beginning from an accessible agricultural waste product, making this material appealing in the search for nonhazardous, environmentally benign antimicrobial materials. To this extent, naturally derived alternatives to the polymer support may also be explored in the future to further the appeal of this material as a green alternative and expand its potential applications [49].

Overall, the synthesis of polymer-embedded AgNP@RHsil takes advantage of the photoinduced transport of AgNP@RHsil towards the surface. This migration of the active antimicrobial material is selective, unidirectional, and leads to a great enhancement in antimicrobial performance. Leaching tests at this initial stage of the development are promising and more in-field studies on the re-usability and long-term durability would be interesting before it reaches the market. The proposed multilayered material, unlike colloidal particles, is amenable to consumer applications

## Data Availability

The original contributions presented in this study are included in the article/Supplementary Materials. Further inquiries can be directed to the corresponding author.

## Supplementary Materials

The following supporting information can be downloaded at: https://www.mdpi.com/article/10.3390/nano15161224/s1, materials preparation, EDS and SEM data, antibacterial set-up and related experiments, ICP studies and selected optical and electron microscopy images.

## References

[1] George C.M., Cirhuza L.B., Kuhl J., Williams C., Coglianese N., Thomas E., Bauler S., Francois R., Saxton R., Presence A.S. (2021). Child Mouthing of Feces and Fomites and Animal Contact are Associated with Diarrhea and Impaired Growth Among Young Children in the Democratic Republic of the Congo: A Prospective Cohort Study (REDUCE Program). J. Pediatr..

[2] Otter J.A., Yezli S., Salkeld J.A.G., French G.L. (2013). Evidence that contaminated surfaces contribute to the transmission of hospital pathogens and an overview of strategies to address contaminated surfaces in hospital settings. Am. J. Infect. Cont..

[3] Chen T. (2020). A Rapid Review of Disinfectant Chemical Exposures and Health Effects During COVID-19 Pandemic.

[4] Salonen H., Salthammer T., Castagnoli E., Taubel M., Morawska L. (2024). Cleaning products: Their chemistry, effects on indoor air quality, and implications for human health. Environ. Int..

[5] Parveen N., Chowdhury S., Goel S. (2022). Environmental impacts of the widespread use of chlorine-based disinfectants during the COVID-19 pandemic. Environ. Sci. Pollut. Res. Int..

[6] Jose A., Gizdavic-Nikolaidis M., Swift S. (2023). Antimicrobial Coatings: Reviewing Options for Healthcare Applications. Appl. Microbiol..

[7] Barrocas B.T., Fernandes S.M., Alcobia T., Lourenço M.C., Oliveira M.C., Marques A.C. (2025). Optimization of TiO_2_ loaded sol-gel derived MICROSCAFS^®^ for enhanced minocycline removal from water and real wastewater. J. Sol.-Gel Sci. Technol..

[8] Blaskovicova J., Labuda J. (2022). Effect of Triclosan and Silver Nanoparticles on DNA Damage Investigated with DNA-Based Biosensor. Sensors.

[9] Langiano F., Fernandes S.M., Barrocas B.T., Del Tedesco A., Riello P., Ferreira M.J., Marques A.C., Sgarzi M., Gigli M., Crestini C. (2025). Rice husk silica derived MICROSCAFS^®^ for a green solar-driven photodegradation of minocycline in aqueous media. J. Water Proc. Eng..

[10] Attol D.H., Mihsen H.H., Jaber S.A., Alwazni W.S., Eesa M.T. (2023). Synthesis of Organic Functionalized Silica from Rice Husk as an Antibacterial Agents. Silicon.

[11] Alhadhrami A., Mohamed G.G., Sadek A.H., Ismail S.H., Ebnalwaled A.A., Almalki A.S.A. (2022). Behavior of Silica Nanoparticles Synthesized from Rice Husk Ash by the Sol–Gel Method as a Photocatalytic and Antibacterial Agent. Materials.

[12] Hohm T., Preuten T., Fankhauser C. (2013). Phototropism: Translating light into directional growth. Am. J. Bot..

[13] Frank C.J., Bourgonje C.R., Yaghmaei M., Scaiano J.C. (2025). A color-coordinated approach to the flow synthesis of silver nanoparticles with custom morphologies. Nanoscale Adv..

[14] Shrestha D., Nayaju T., Kandel M.R., Pradhananga R.R., Park C.H., Kim C.S. (2023). Rice husk-derived mesoporous biogenic silica nanoparticles for gravity chromatography. Heliyon.

[15] Popova M., Mitova V., Dimitrov M., Rosmini C., Tsacheva I., Shestakova P., Karashanova D., Karadjova I., Koseva N. (2024). Mesoporous Silica with an Alveolar Construction Obtained by Eco-Friendly Treatment of Rice Husks. Molecules.

[16] Schindelin J., Arganda-Carreras I., Frise E., Kaynig V., Longair M., Pietzsch T., Preibisch S., Rueden C., Saalfeld S., Schmid B. (2012). Fiji: An open-source platform for biological-image analysis. Nat. Meth..

[17] Bourgonje C.R., da Silva D.R.C., McIlroy E., Calvert N.D., Shuhendler A.J., Scaiano J.C. (2023). Silver nanoparticles with exceptional near-infrared absorbance for photoenhanced antimicrobial applications. J. Mater. Chem. B.

[18] Chang B.M., Pan L., Lin H.H., Chang H.C. (2019). Nanodiamond-supported silver nanoparticles as potent and safe antibacterial agents. Sci. Rep..

[19] Kim D., Kwon S.J., Wu X., Sauve J., Lee I., Nam J., Kim J., Dordick J.S. (2018). Selective Killing of Pathogenic Bacteria by Antimicrobial Silver Nanoparticle-Cell Wall Binding Domain Conjugates. ACS Appl. Mater. Interf..

[20] Paramesh C.C., Giridasappa A., Siddegowda A.K.C., Rangappa D., Shivaramu P.D. (2024). History, introduction, and physicochemical properties of silver nanoparticles. Silver Nanoparticles for Drug Delivery.

[21] Li H., Xu H. (2024). Mechanisms of bacterial resistance to environmental silver and antimicrobial strategies for silver: A review. Environ. Res..

[22] Zhang L., Zhao Y., Lin Z., Gu F., Lau S.P., Li L., Chai Y. (2015). Kinetically controlled synthesis of large-scale morphology-tailored silver nanostructures at low temperature. Nanoscale.

[23] Bai W., Liang B., Luo B., Wang J., Zhang H., Zhang X., Yang L., Xu Y., Li Y. (2025). Ultra-High Bromine Removal from Waste Water and Downstream High-Value Utilization Using Melanin-Like Polymers. Small.

[24] Yang Y., Yang L., Yang F., Bai W., Zhang X., Li H., Duan G., Xu Y., Li Y. (2023). A bioinspired antibacterial and photothermal membrane for stable and durable clean water remediation. Mater. Horiz..

[25] Adam F., Appaturi J.N., Iqbal A. (2012). The utilization of rice husk silica as a catalyst: Review and recent progress. Catal. Today.

[26] Pastor A., Balbuena J., Cruz-Yusta M., Pavlovic I., Sánchez L. (2019). ZnO on rice husk: A sustainable photocatalyst for urban air purification. Chem. Eng. J..

[27] He D., Ikeda-Ohno A., Boland D.D., Waite T.D. (2013). Synthesis and characterization of antibacterial silver nanoparticle-impregnated rice husks and rice husk ash. Environ. Sci. Technol..

[28] Ghobadi M., Salehi S., Ardestani M.T.S., Mousavi-Khattat M., Shakeran Z., Khosravi A., Cordani M., Zarrabi A. (2024). Amine-functionalized mesoporous silica nanoparticles decorated by silver nanoparticles for delivery of doxorubicin in breast and cervical cancer cells. Eur. J. Pharm. Biopharm..

[29] Mierina I., Grigale-Sorocina Z., Birks I. (2025). The Chemistry of Behind the UV-Curable Nail Polishes. Polymers.

[30] Ashfaq A., Clochard M.-C., Coqueret X., Dispenza C., Driscoll M.S., Ulański P., Al-Sheikhly M. (2020). Polymerization Reactions and Modifications of Polymers by Ionizing Radiation. Polymers.

[31] Dube E., Okuthe G.E. (2025). Silver Nanoparticle-Based Antimicrobial Coatings: Sustainable Strategies for Microbial Contamination Control. Microbiol. Res..

[32] Barrocas B., Nunes C.D., Carvalho M.L., Monteiro O.C. (2016). Titanate nanotubes sensitized with silver nanoparticles: Synthesis, characterization and in-situ pollutants photodegradation. Appl. Surf. Sci..

[33] Nzereogu P.U., Omah A.D., Ezema F.I., Iwuoha E.I., Nwanya A.C. (2023). Silica extraction from rice husk: Comprehensive review and applications. Hybrid Adv..

[34] Stamplecoskie K.G., Scaiano J.C., Tiwari V.S., Anis H. (2011). Optimal Size of Silver Nanoparticles for Surface-Enhanced Raman Spectroscopy. J. Phys. Chem. C.

[35] Stamplecoskie K.G., Scaiano J.C. (2010). Light Emitting Diode Irradiation Can Control the Morphology and Optical Properties of Silver Nanoparticles. J. Am. Chem. Soc..

[36] Tien C.C., Chang C.H., Liu B.H., Stanley D., Rabb S.A., Yu L.L., Nguyen T., Sung L. Effects of temperature on surface accumulation and release of silica nanoparticles in an epoxy nanocoating exposed to UV radiation. Proceedings of the Technical Proceedings of the 2014 NSTI Nanotechnology Conference and Expo, NIST-Nanotech 2014.

[37] Haassan A.O., Ojo B.O., Abdulrahman A.O. (2021). Escherichia coli as a Global Pathogen. Achiev. J. Sci. Res..

[38] Pokharel P., Dhakal S., Dozois C.M. (2023). The Diversity of Escherichia coli Pathotypes and Vaccination Strategies against This Versatile Bacterial Pathogen. Microorganisms.

[39] Silvero M.J., Argüello G.A., Becerra M.C. (2014). Photodynamic Antibacterial Chemoterapy (PACT) Using Gold Nanoparticles and LED’s Irradiation. J. Nanopharm. Drug Del..

[40] Yougbare S., Mutalik C., Krisnawati D.I., Kristanto H., Jazidie A., Nuh M., Cheng T.M., Kuo T.R. (2020). Nanomaterials for the Photothermal Killing of Bacteria. Nanomaterials.

[41] Dong W., Huang L., Song X., Zhang Y., Liu M., Ren Z., Pang L., Peng H., Jiang H. (2024). Carbon nanotube-based photothermal membrane for efficient cold air heating and removal of particulate matter and airborne bacteria. Green Carbon.

[42] Silvero M.J., Becerra M.C. (2016). Plasmon-induced oxidative stress and macromolecular damage in pathogenic bacteria. RSC Adv..

[43] Cao J., Song Z., Du T., Du X. (2024). Antimicrobial materials based on photothermal action and their application in wound treatment. Burns Trauma.

[44] Santos G.M., Ferrara F.I.d.S., Zhao F., Rodrigues D.F., Shih W.-C. (2016). Photothermal inactivation of heat-resistant bacteria on nanoporous gold disk arrays. Opt. Mater. Express.

[45] Yaghmaei M., Bourgonje C.R., Scaiano J.C. (2023). Facile Scale-Up of the Flow Synthesis of Silver Nanostructures Based on Norrish Type I Photoinitiators. Molecules.

[46] (2024). Performance Standards for Antimicrobial Susceptibility Testing, 34th ed.

[47] (2011). Measurement of Antibacterial Activity on Plastics and Other Non-Porous Surfaces, 2nd ed.

[48] (2021). Evaluation of Methods for Assessing the Release of Nanomaterials from Commercial, Nanomaterial-Containing Polymer Composites.

[49] Zhang Y., Deng W., Wu M., Yu G., Liu Z., Cheng N., Du H., Liu C., Li B. (2024). Engineering pulp foam with highly improved water stability and multifunctional properties by incorporation of natural rubber and montmorillonite. Green Carbon.

