# Supplementary material for: Photoinduced Transport and Activation of Polymer-Embedded Silver on Rice Husk Silica Nanoparticles for a Reusable Antimicrobial Surface
Source: Nanomaterials (Basel). 2025 Aug 11;15(16):1224. doi: 10.3390/nano15161224 (PMC12388695; doi:10.3390/nano15161224)
Supplement: Supplementary file 1 [file nanomaterials-15-01224-s001.zip › nanomaterials-3781230-supplementary.pdf]

Supplementary Material for

Photoinduced transport and activation of polymer-embedded silver on rice husk silica nanoparticles for a reusable antimicrobial surface

Carly J. Frank, Vivian He, Juan C. Scaiano, and M. Jazmin Silvero C.

Department of Chemistry and Biomolecular Sciences, University of Ottawa, Ottawa, Ontario, K1N 6N5, Canada

Table of Contents

RHSIL AND ANTIMICROBIAL MATERIAL PREPARATION 2
EDS DATA FOR MAIN TEXT FIGURES 7
SELECTED SEM IMAGES OF MATERIAL LAYERS 9
EDS REPORTS OF MULTILAYER MATERIAL CROSS SECTIONS 12
VISIBLE LIGHT ANTIMICROBIAL ACTIVITY – EXPERIMENTAL SETUP 15
MULTILAYER MATERIAL COMPONENTS 16
COMPLETE ANTIBACTERIAL PERFORMANCE RESULTS OF MATERIAL OVER SEVERAL USES 17
SELECTED SEM IMAGES OF MATERIAL AFTER EXPOSURE TO *E. COLI* 18
ICP DETECTION OF LEACHED SILVER AND SILICA NANOPARTICLES AFTER REPEATED USE OF THE MATERIAL 21
Table S2a: Initial sample, freshly made 21
Table S2b: Sample used in one test 21
Table S2c: Sample used 8 times 22
IRRADIANCE OF KESSIL LAMP THROUGH PLASTIC MICROSCOPE SLIDE 23

RHsil and antimicrobial material preparation

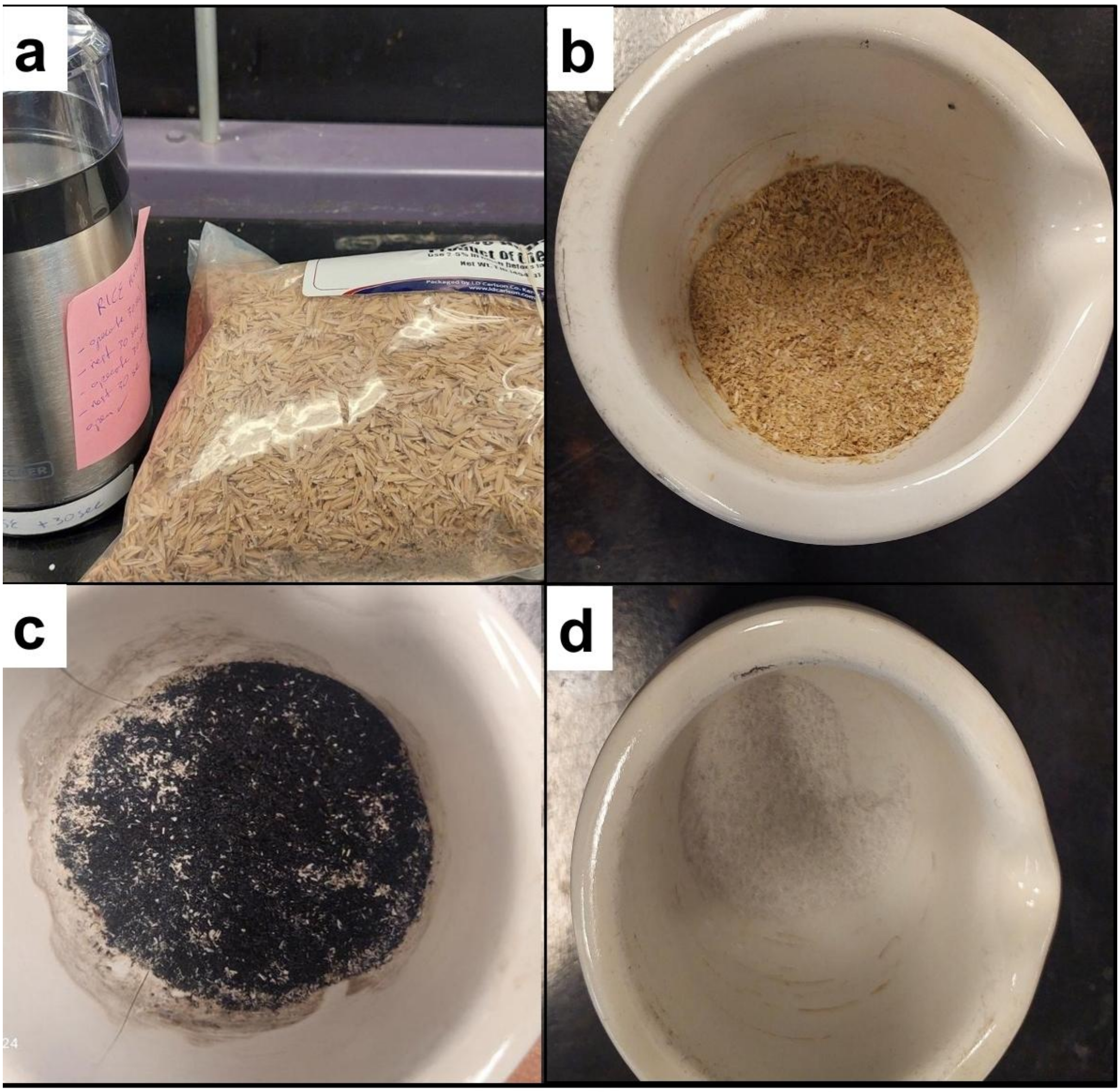

Figure S1. Selected pictures of RHsil preparation: (a) RH as purchased from Toronto Brewing Company next to the coffee grinder machine used; (b) RH fine powder after grinding; (c) RH after 1.5 h of calcination, referred as rice husk black ash in the main text; (d) RHsil white ash after 3 h of calcination.

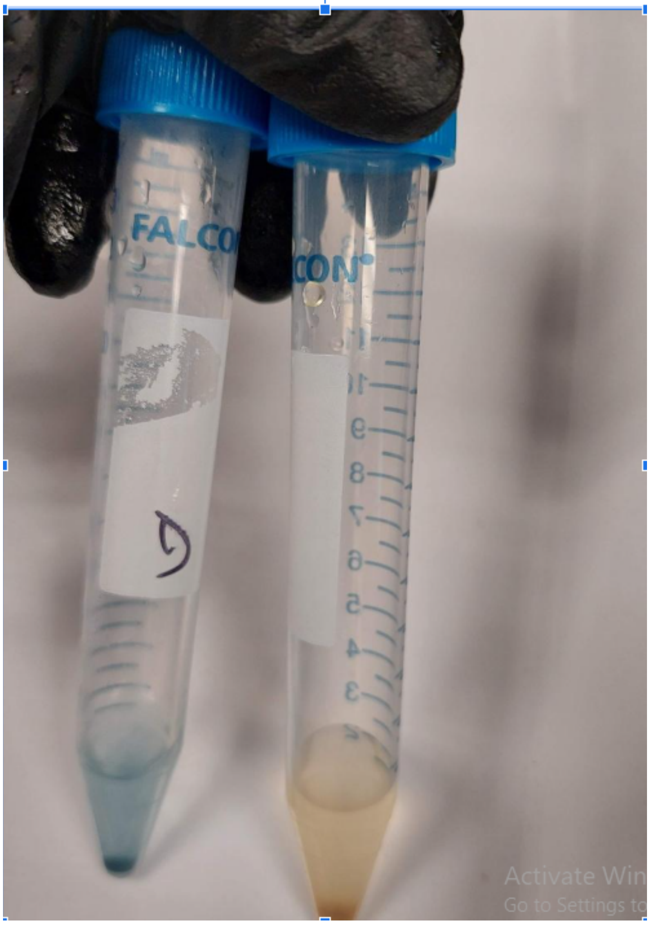

Figure S2. Resulting nanoparticles after mixing RHsil with tAgNP (light blue sample on the left) and AgNP seeds (yellow sample on the right). For this work only the triangle nanoparticles on RHsil.

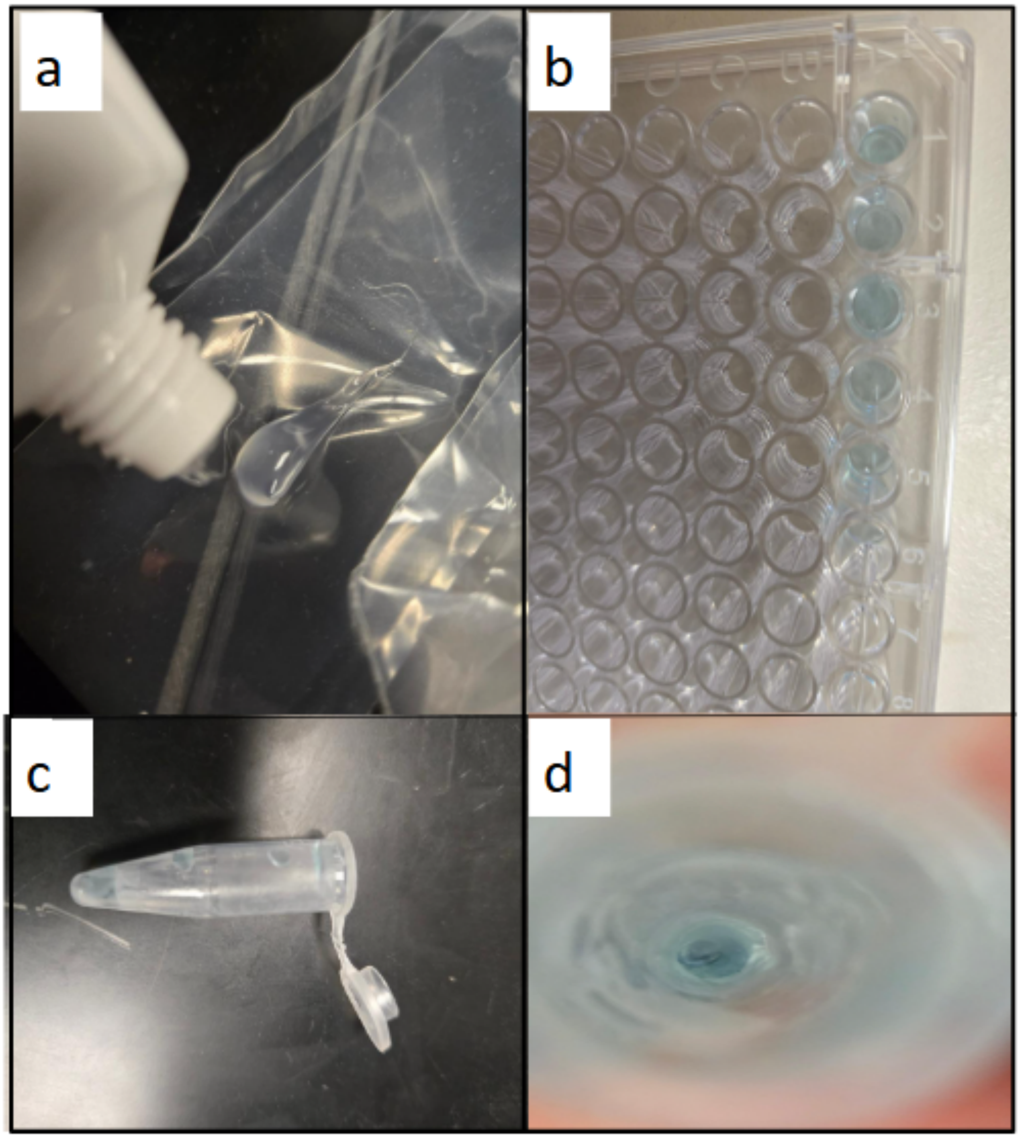

Figure S3. Selected pictures of the mixing process of AgNP@RHsil directly with the methacrylate gel: (a) Colorless and sticky nail gel right out of the commercial container; (b) Top view of the 96 well plate used for the antimicrobial experiments; (c) Close up picture of the mixture inside an Eppendorf; (d) Close up of the bottom of a well containing the unsolidified mixture after the attempt to crosslink with UVA.

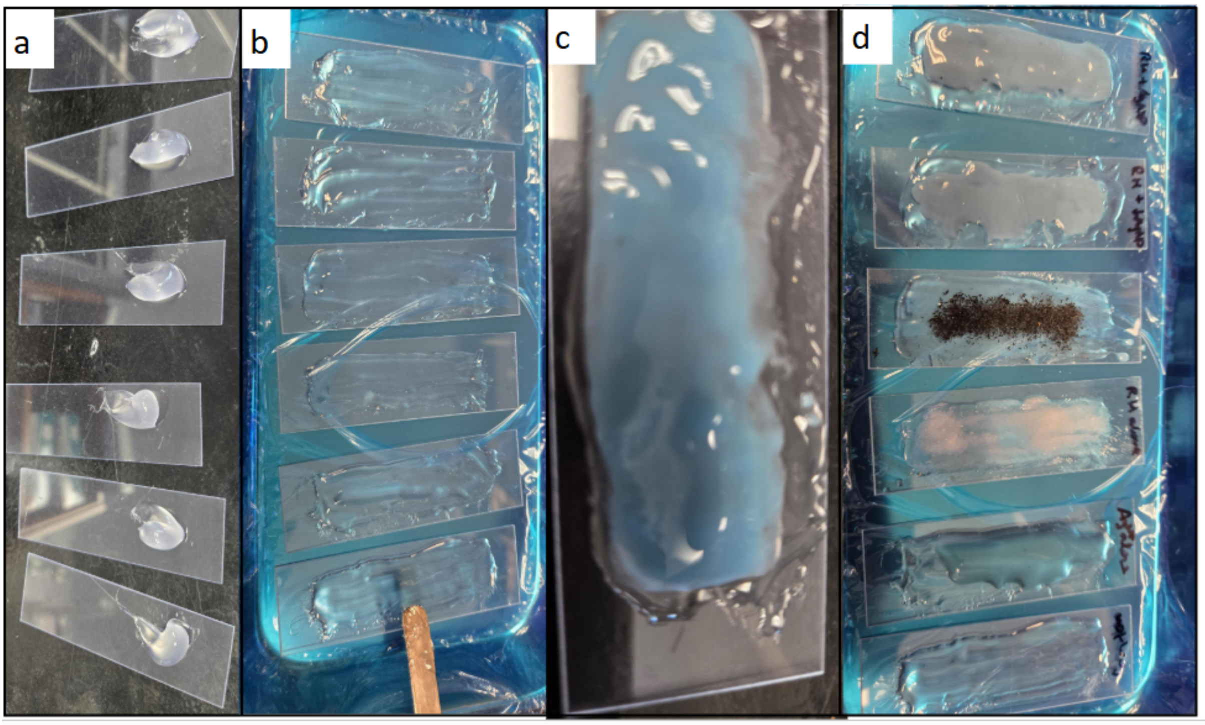

Figure S4. Pictures of the sandwich preparation steps. (a) Gel portions (3 g) placed in plastic microscope slides, (b) Gel spread on six microscope slides before UVA exposure, (c) AgNP@RHsil light blue slurry spread on top of the first layer of gel already solidified, (d) Microscope slides with the first layer of polymer crosslinked and different samples spread on top: AgNP@RHsil (duplicates), rice husk black ashes, RHsil, AgNP, ultrapure water (polymer alone), from top to bottom, used as controls in the antibacterial assessment

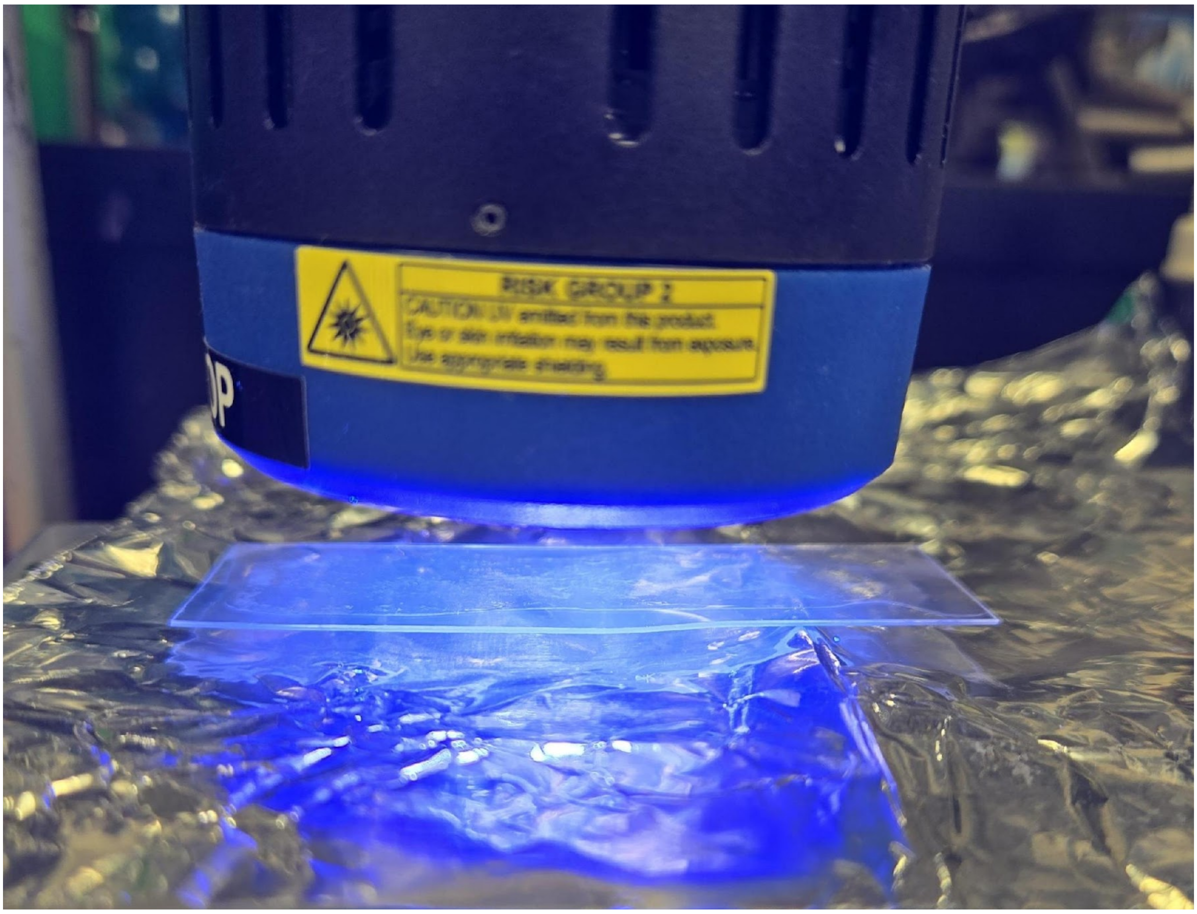

Figure S5. Picture of the crosslinking setup for the gel layers of the material. A Kessil lamp placed at 2 cm distance from the slide provided the UVA light.

EDS data for main text figures

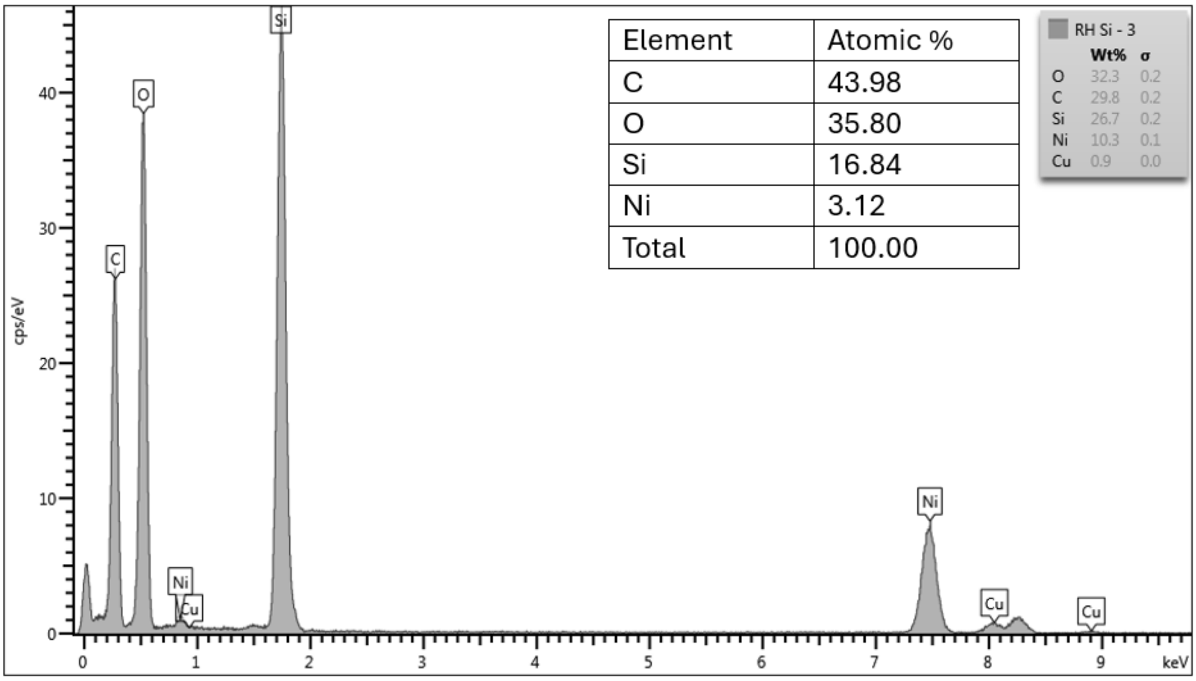

Figure S6. EDS report of Figure 2b in the main text.

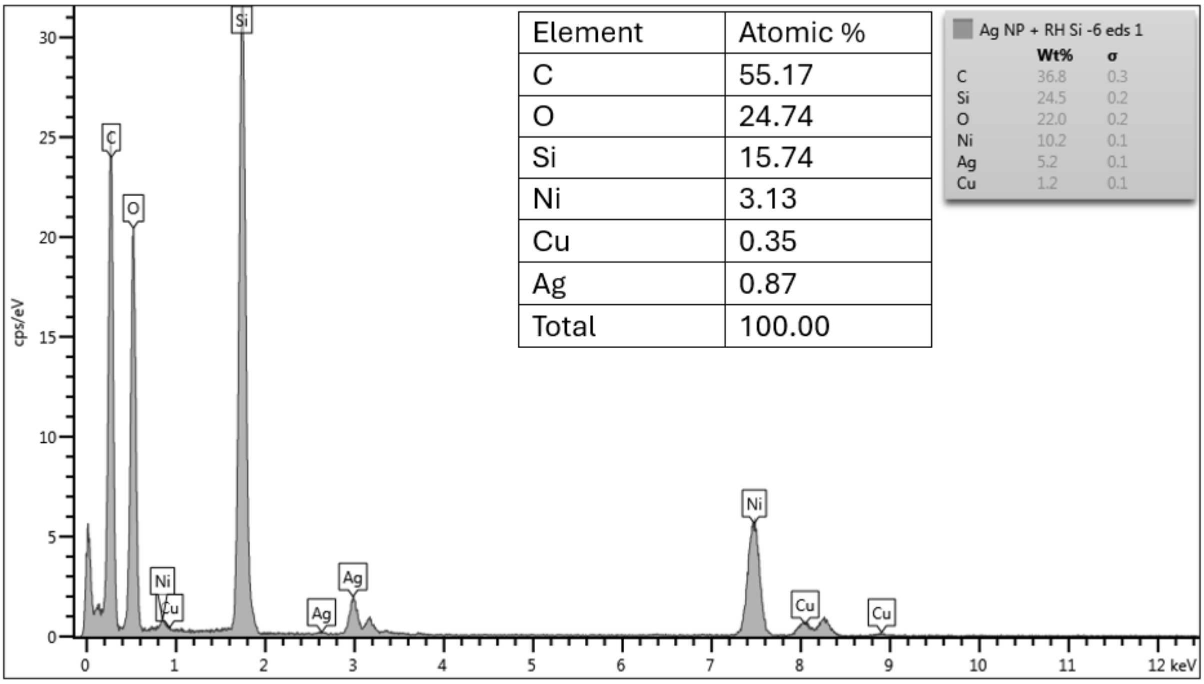

Figure S7. EDS report of Figure 2c in the main text.

Selected SEM images of material layers

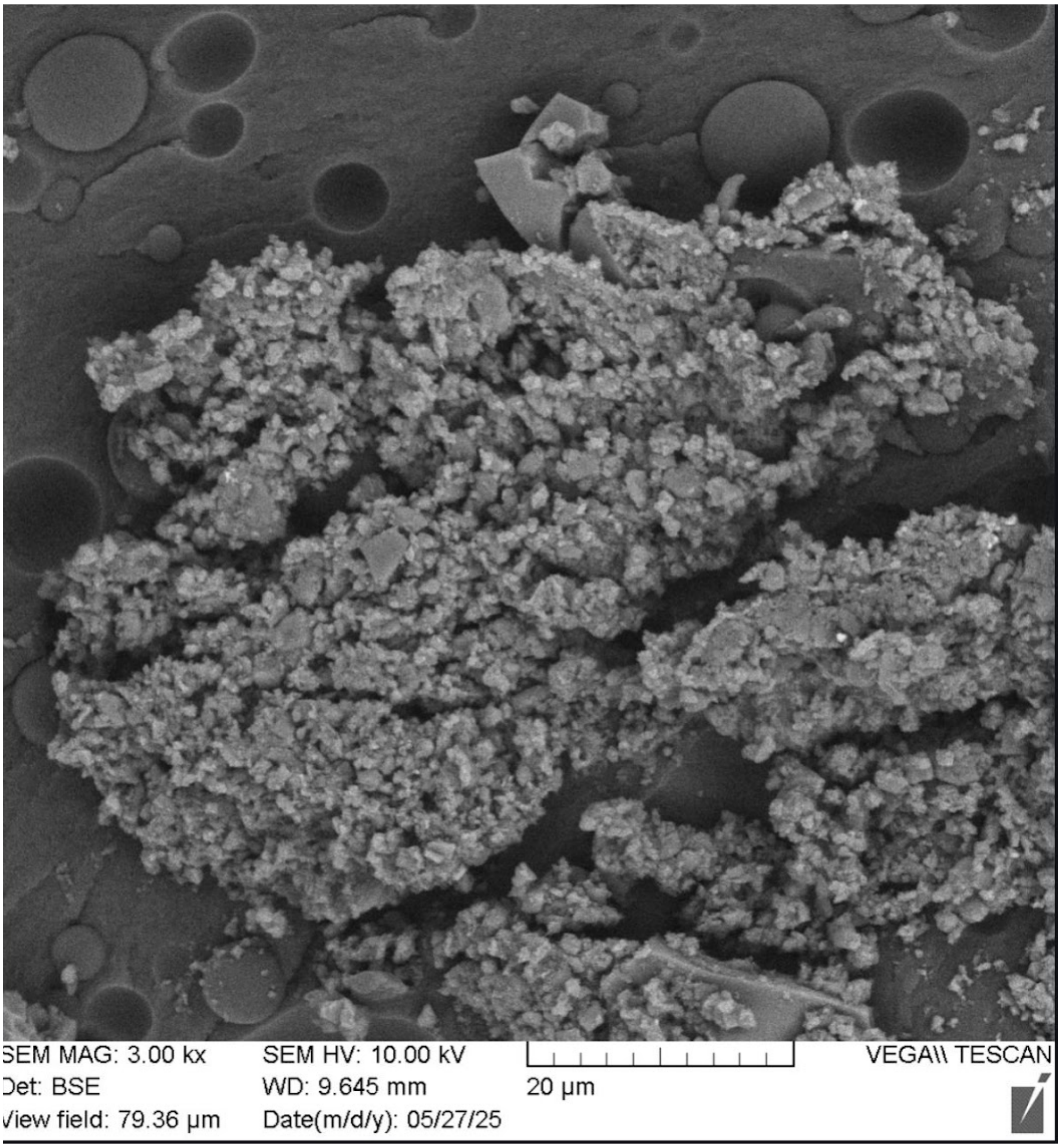

Figure S8. Uncropped SEM of multilayered material, the middle layer showing the nanoparticle filling.

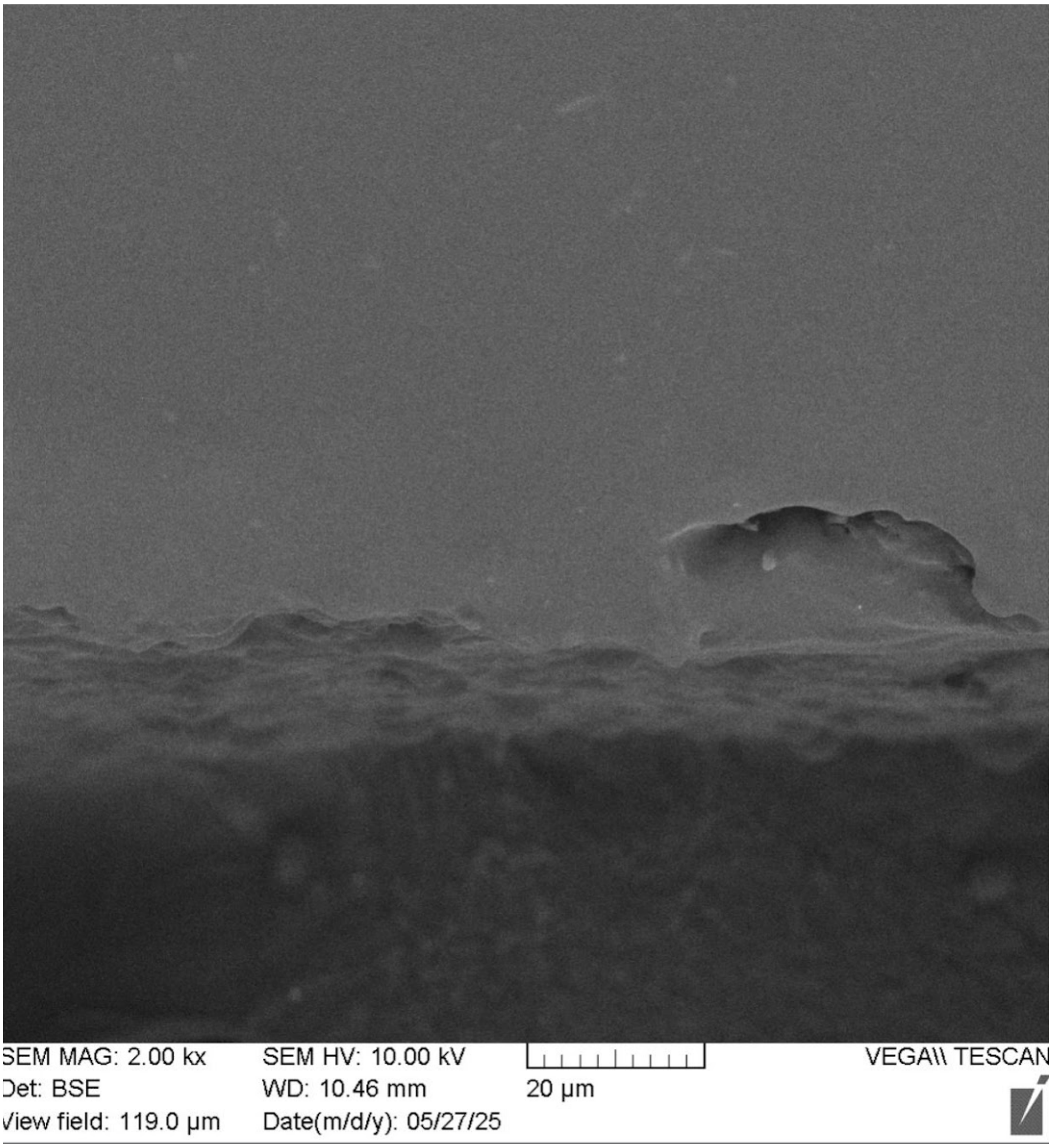

Figure S19. Uncropped SEM of multilayered material, the bottom layer showing a smooth polymer without nanoparticles.

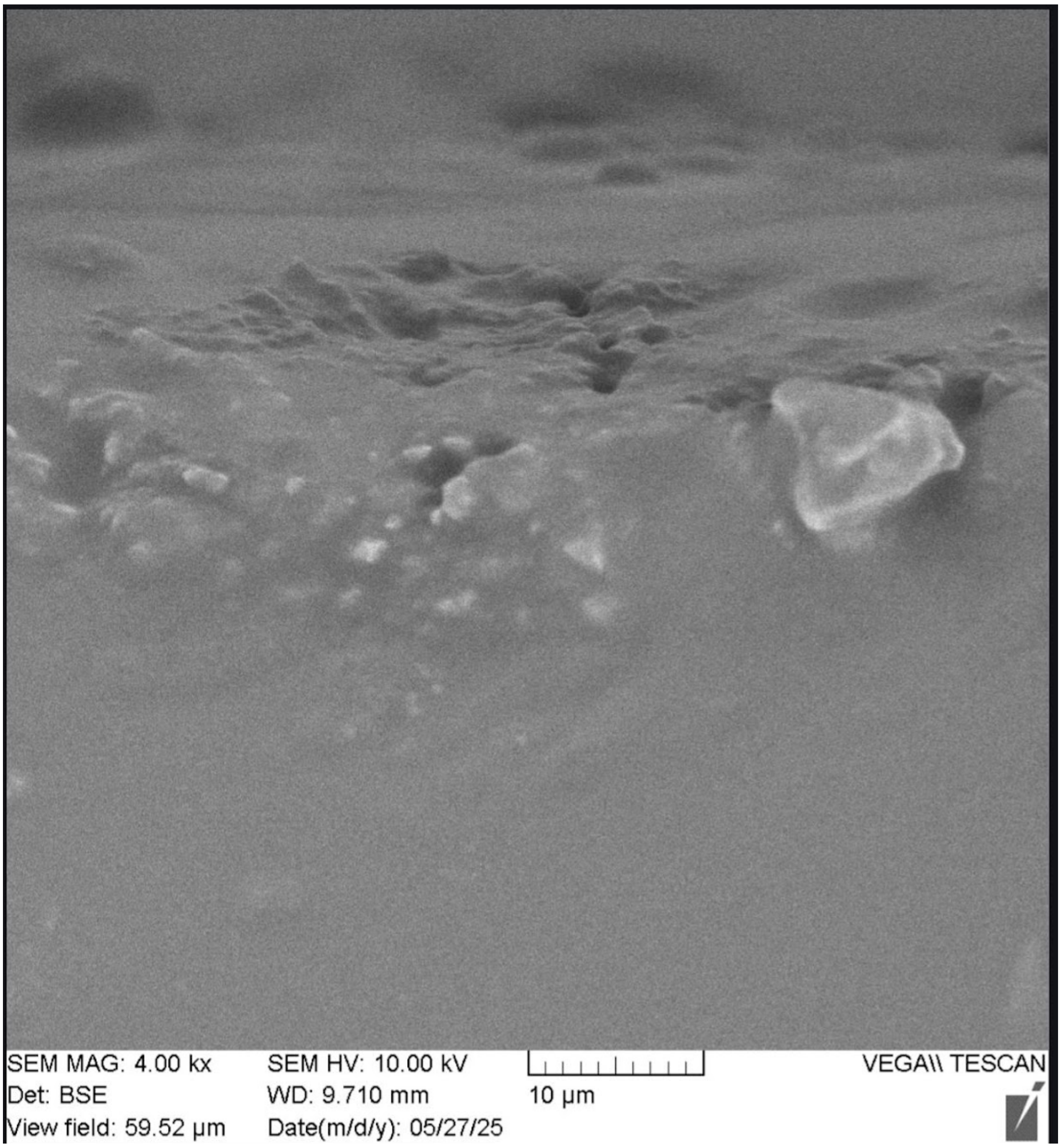

Figure S10. Uncropped SEM of multilayered material, the top layer showing nanoparticles photo-transported to the surface from the middle layer.

EDS reports of multilayer material cross sections

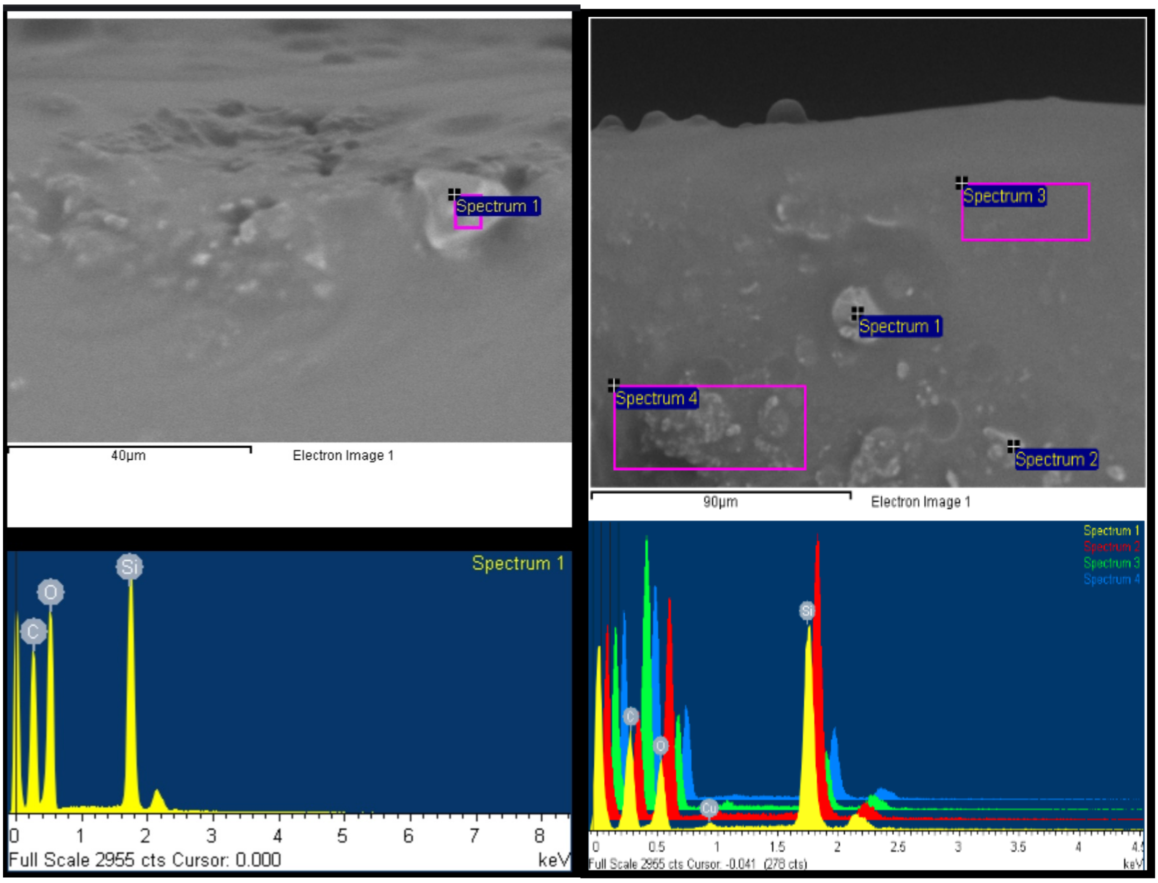

Figure S11. Selected SEM and EDS reports of SiO_2 nanoparticles close to the surface of the top polymer layer, corresponding to Figure 3b in the main text. AgNP are assumed to be inside, but the probe is not sensitive enough to detect them.

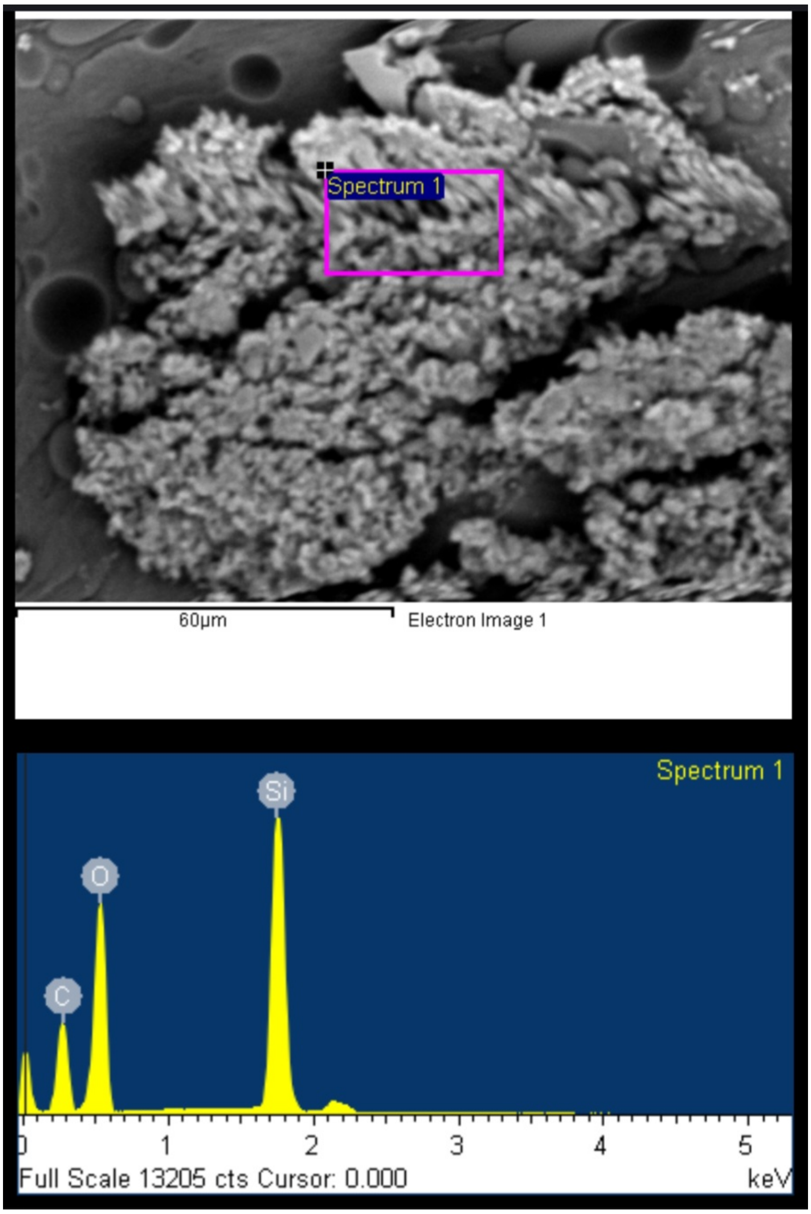

Figure S12. Selected SEM and EDS report of middle layer corresponding with AgNP@RHsil, corresponding to Figure 3c in the main text. The AgNP are assumed to be inside, but the probe is not sensible enough to detect them.

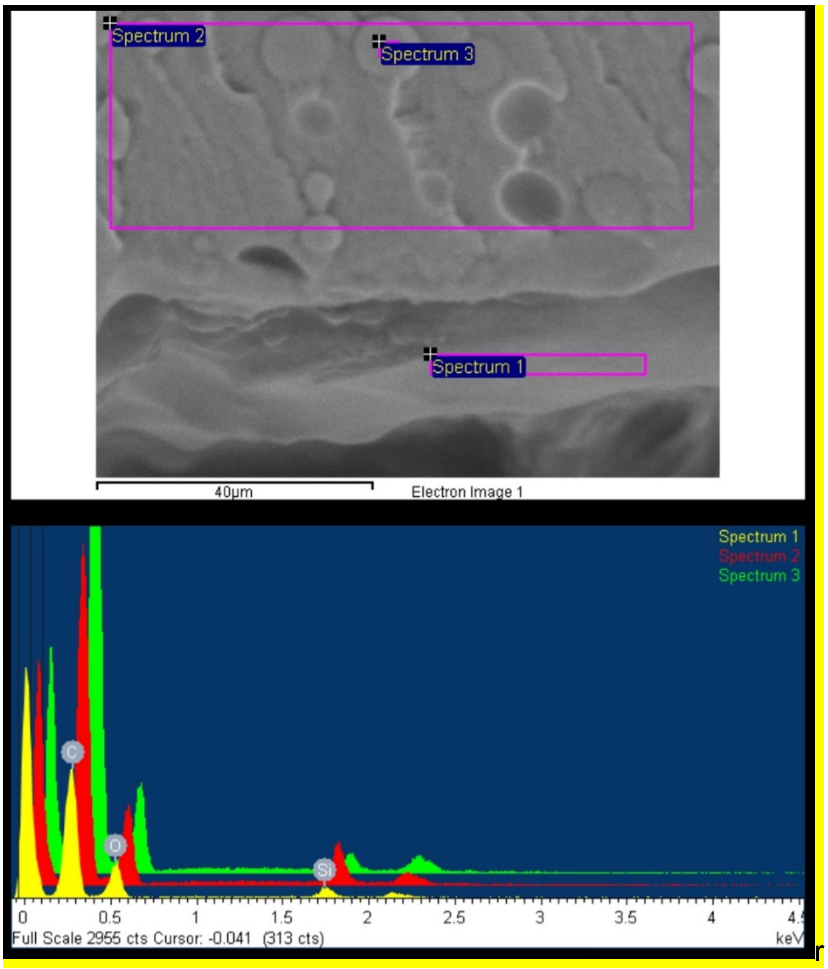

Figure S13. Selected SEM and EDS report of nanoparticle-free bottom layer of polymer, corresponding to Figure 3a in the main text. The infimal amount of silica detected corresponds to a component of the commercial gel nail.

Visible light antimicrobial activity – experimental setup

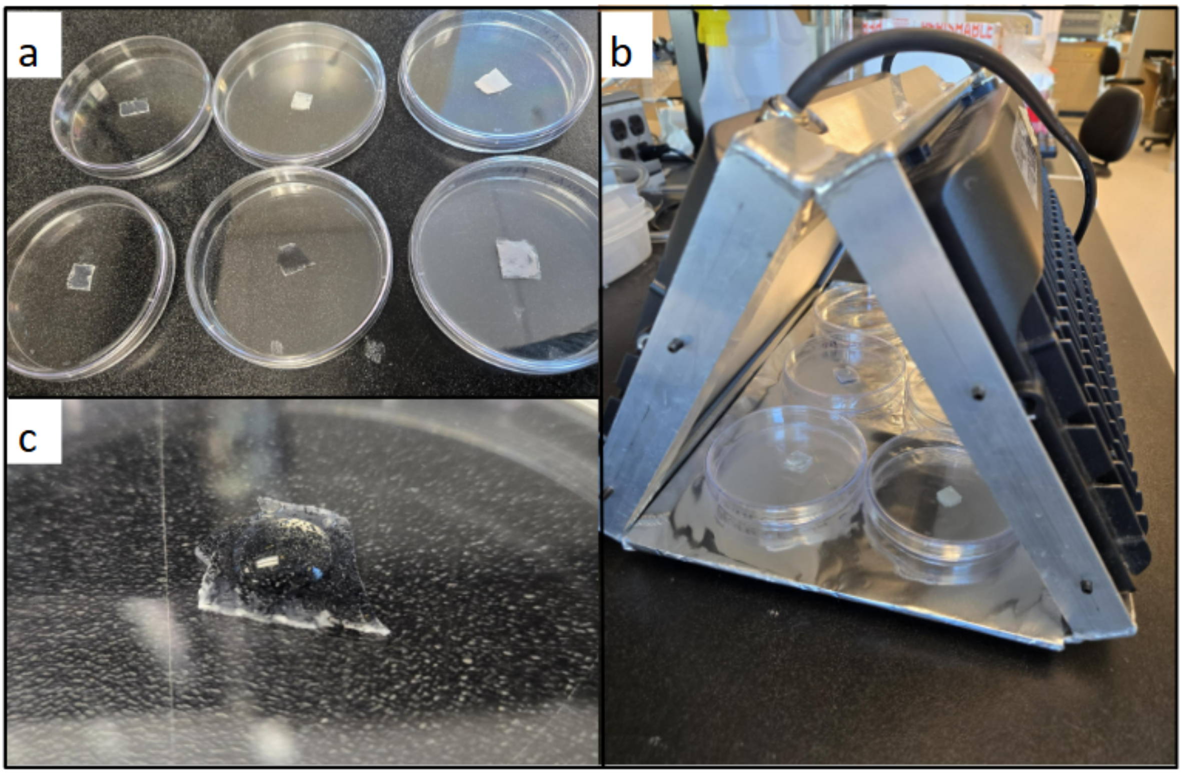

Figure S14. Selected pictures of the antimicrobial test experimental set up for the multilayered material according to ISO 22196: (a) Petri dishes containing different square pieces of the surface to be tested; (b) White LED panels placed in a tent-like arrangement over 6 Petri dishes at the time for irradiation, with aluminum foil underneath the samples; (c) Close-up of bacterial inoculum drop on the surface of a sandwich filled with rice husk black ashes as control.

Multilayer material components

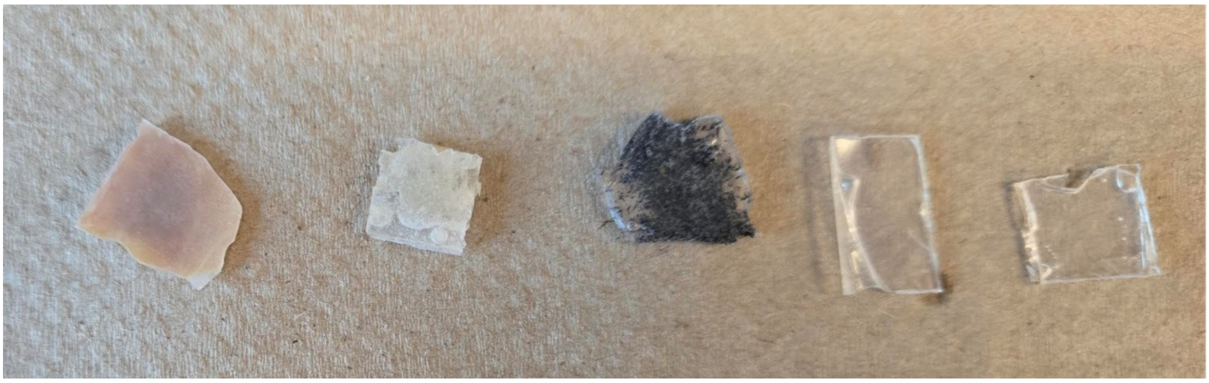

Figure S15. Pieces of multilayered polymer containing (from left to right): AgNP@RHsil, RHsil, RH black ashes, AgNP and polymer alone, after fixation process for SEM. Surface remains intact. Duplicates for irradiated samples and controls kept in darkness were analyzed the same day. Bacterial growth was found in all samples with exemption of the sandwich containing AgNP@RHsil irradiated, proving that neither of the components alone nor the light by itself have antimicrobial capabilities. A selected SEM of the polymer alone is included below as an example.

Complete antibacterial performance results of material over several uses

Table S1. Antibacterial performance in *E. coli* of layered materials after 10, 20, and 30 minutes without (Dark) and with (Light) irradiation under visible light. Numbers are expressed as Log reduction of bacterial growth in CFU/mL compared to the initial inoculum CFU/mL provided in brackets. Average log reductions and their standard deviations are provided in the last row. Controls of bacteria on polymer alone, and sandwiches filled with not photo-induced migrated of AgNP@RHsil, white rice husk ashes, black rice husk ashes, and silver triangles nanoplates alone did not show significant modification in bacterial growth at any time even under irradiation (LOG reduction <0.1) and were not included in this graphic (LOG reduction between 0 and 0.1). These were not included in the table for space reasons.

	10 minutes		20 minutes		30 minutes	
Use	Dark	Light	Dark	Light	Dark	Light
1	0.2 (5.5)	5.4 (5.5)	0.2 (5.5)	5.5 (5.5)	-0.2 (5.5)	5.4 (5.5)
2	0.1 (5.5)	5.5 (5.5)	0.2 (5.5)	5.5 (5.5)	0 (5.5)	5.5 (5.5)
3	0 (6)	6 (6)	0 (6)	6 (6)	-0.1 (6)	6 (6)
4	0.2 (5.8)	5.6 (5.8)	0.3 (5.8)	5.8 (5.8)	0.2 (5.8)	5.8 (5.8)
5	0.2 (5.6)	5.6 (5.6)	0 (5.6)	5.5 (5.6)	0 (5.6)	5.6 (5.6)
6	0 (5.6)	5.5 (5.5)	-0.2 (5.5)	5.5 (5.5)	-0.2 (5.5)	5.5 (5.5)
7	0 (5.9)	5.9 (5.9)	0.1 (5.9)	5.9 (5.9)	0 (5.9)	5.9 (5.9)
8	0.1 (5.5)	5.5 (5.5)	0 (5.5)	5.5 (5.5)	-0.2 (5.5)	5.5 (5.5)
AVERAGE	0.10 ± 0.09	5.6 ± 0.2	0.1 ± 0.2	5.6 ± 0.2	-0.1 ± 0.1	5.6 ± 0.2

Selected SEM images of material after exposure to *E. coli*

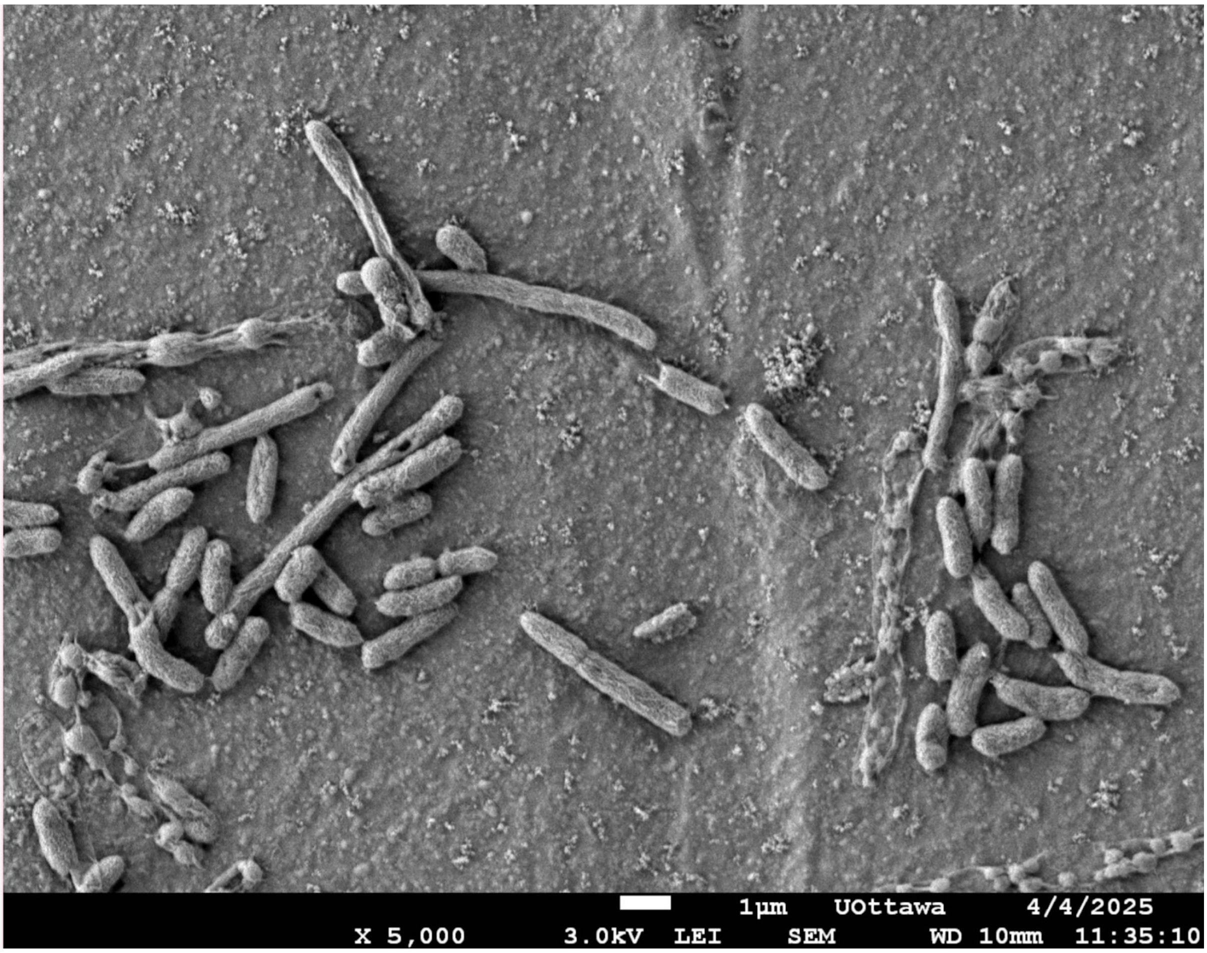

Figure S16. Uncropped SEM of pizza-like material kept in the dark showing *Escherichia coli* growth.

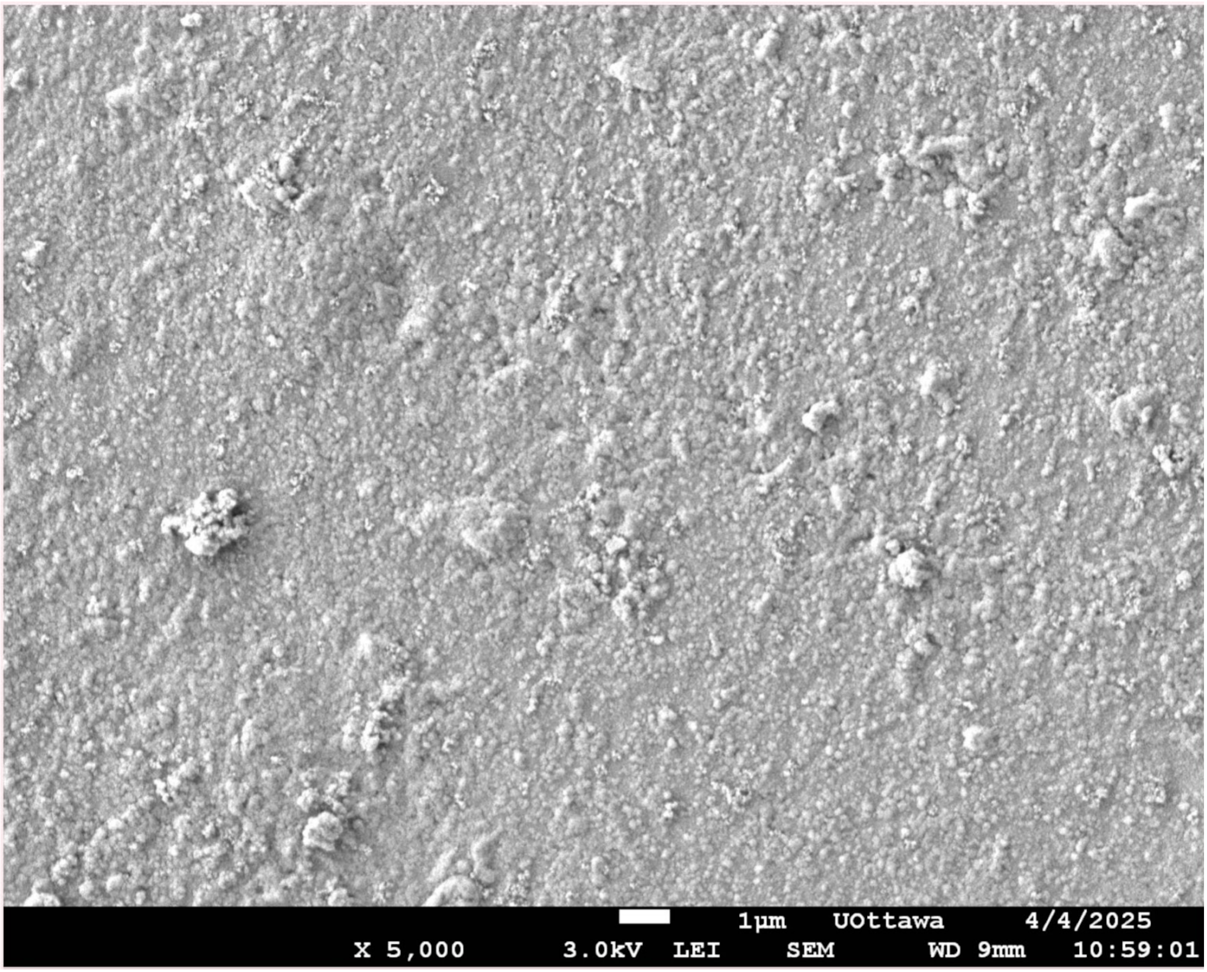

Figure S17. Uncropped SEM of pizza-like material exposed to bacteria, after LED irradiation showing the absence of microorganisms.

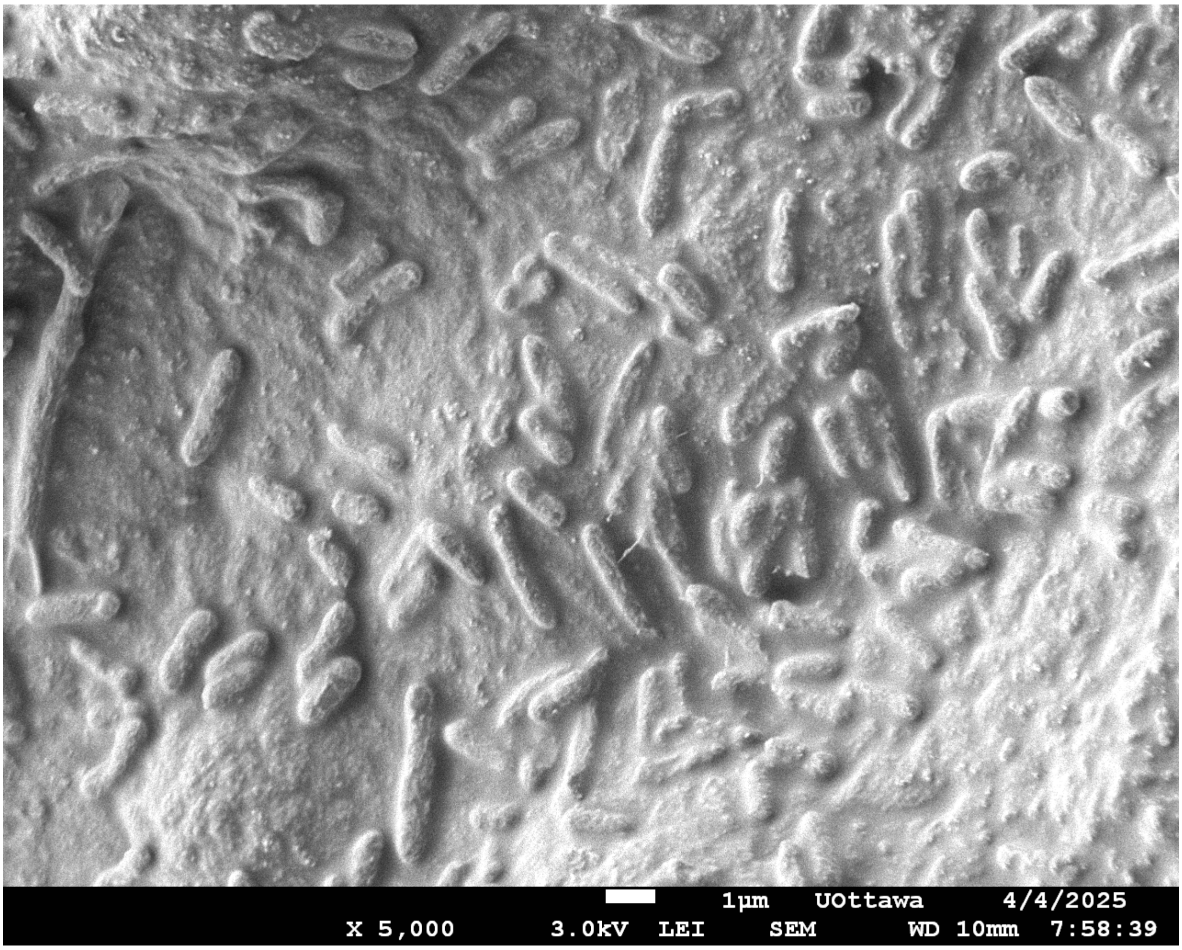

Figure S18. SEM of polymer alone exposed to bacteria, after LED irradiation showing the integrity of microorganisms.

ICP detection of leached silver and silica nanoparticles after repeated use of the material

Table S2. Screenshots of the ICP report for the detection of silver and silica in collected samples from the top surface of freshly made multilayered material (a), and after use in antimicrobial experiments 1 (b) and 8 (c) times. A calibration curve was run for silver with the proper standard. Silica could not be quantified but was also not detected at all in any sample but the initial control.

Table S2a: Initial sample, freshly made

Sample Name: Just made

Date: 2025-06-26 11:58:34 PM Rack:Tube: 1:8

Weight (g): 1 Volume (mL): 1 Dilution: 1

Analyte Results

Label	Solution Concentration	Unit	SD	%RSD	Intensity	Calculated Concentration
Ag (328.068 nm)	0.08	ppm	0.00	4.56	1102.37	0.08 (ppm)
Si (251.611 nm)	Uncal	ppm	N/A	N/A	38.53	Uncal (ppm)

Replicates Concentration

Label	Replicate 1	Replicate 2	Replicate 3	Units
Ag (328.068 nm)	0.08	0.08	0.08	ppm
Si (251.611 nm)	Uncal	Uncal	Uncal	ppm

Replicates Intensity

Label	Replicate 1 (c/s)	Replicate 2 (c/s)	Replicate 3 (c/s)
Ag (328.068 nm)	1159.23	1074.61	1073.27
Si (251.611 nm)	41.56	38.24	35.79

Table S2b: Sample used in one test

Sample Name: used 1 time

Date: 2025-06-27 12:00:07 AM Rack:Tube: 1:9

Weight (g): 1 Volume (mL): 1 Dilution: 1

Analyte Results

Label	Solution Concentration	Unit	SD	%RSD	Intensity	Calculated Concentration
Ag (328.068 nm)	0.08	ppm	0.00	2.45	1047.41	0.08 (ppm)
Si (251.611 nm)	Uncal	ppm	N/A	N/A	33.27	Uncal (ppm)

Replicates Concentration

Label	Replicate 1	Replicate 2	Replicate 3	Units
Ag (328.068 nm)	0.08	0.07	0.07	ppm
Si (251.611 nm)	Uncal	Uncal	Uncal	ppm

Replicates Intensity

Label	Replicate 1 (c/s)	Replicate 2 (c/s)	Replicate 3 (c/s)
Ag (328.068 nm)	1076.38	1031.42	1034.43
Si (251.611 nm)	30.71	34.71	34.39

Table S2c: Sample used 8 times

Sample Name: 8 times

Date: 2025-06-27 12:01:40 AM　　Rack:Tube: 1:10

Weight (g): 1　　Volume (mL): 1　　Dilution: 1

Analyte Results

Label	Solution Concentration	Unit	SD	%RSD	Intensity	Calculated Concentration
Ag (328.068 nm)	0.07	ppm	0.00	4.68	1008.22	0.07 (ppm)
Si (251.611 nm)	Uncal	ppm	N/A	N/A	42.37	Uncal (ppm)

Replicates Concentration

Label	Replicate 1	Replicate 2	Replicate 3	Units
Ag (328.068 nm)	0.08	0.07	0.07	ppm
Si (251.611 nm)	Uncal	Uncal	Uncal	ppm

Replicates Intensity

Label	Replicate 1 (c/s)	Replicate 2 (c/s)	Replicate 3 (c/s)
Ag (328.068 nm)	1050.15	958.81	1015.71
Si (251.611 nm)	39.85	46.76	40.49

Irradiance of Kessil lamp through plastic microscope slide

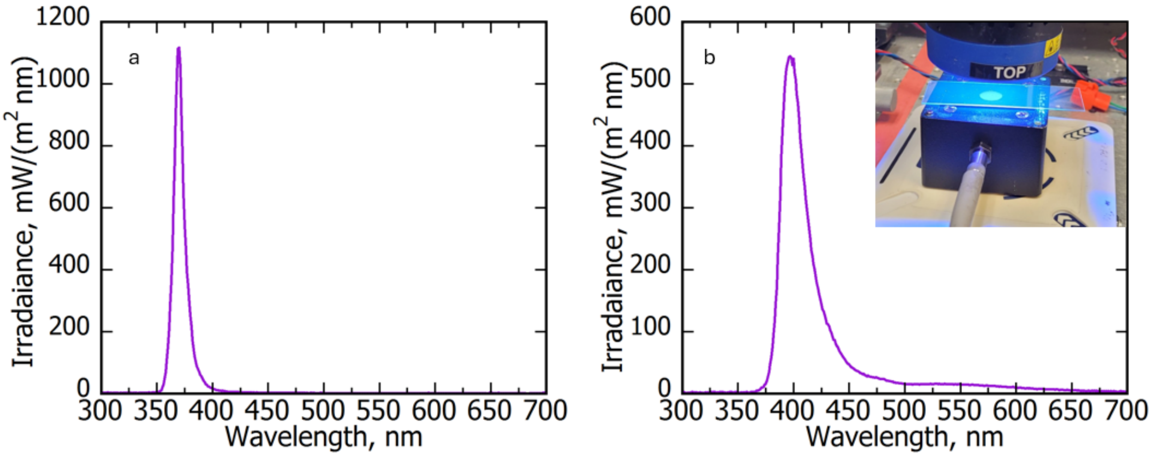

Figure S19. Irradiance measurement of Kessil lamp without and through plastic microscope slide as used for the multilayered material synthesis. The Kessil lamp was placed directly over the slide at a distance of 2 cm at 100 % power, as was used to crosslink the polymer: (a) Emission intensity of Kessil lamp without the slide. Due to its intensity, a 2% filter was placed over the detector. With filter, the measured irradiance from 325 nm to 675 nm was 14978.6 mW/m^2. This corresponds to an actual intensity of 748930 mW/m^2; (b) Irradiance as received by polymer gel and nanoparticle slurry during synthesis, with light from the Kessil lamp filtered and re-emitted by the plastic microscope slide. The measured irradiance from 325 nm to 675 nm was 20547.5 mW/m^2. Inset: measurement setup, with the Kessil light irradiating from above and the microscope slide directly covering the detector.
